# High-throughput image analysis with deep learning captures heterogeneity and spatial relationships after kidney injury

**DOI:** 10.1038/s41598-023-33433-3

**Published:** 2023-04-19

**Authors:** Madison C. McElliott, Anas Al-Suraimi, Asha C. Telang, Jenna T. Ference-Salo, Mahboob Chowdhury, Abdul Soofi, Gregory R. Dressler, Jeffrey A. Beamish

**Affiliations:** 1 Division of Nephrology, Department of Internal Medicine, University of Michigan, 1500 E. Medical Center Drive, SPC 5364, Ann Arbor, MI 48109 USA; 2 Department of Pathology, University of Michigan, Ann Arbor, MI USA

**Keywords:** Nephrology, Kidney diseases, Image processing

## Abstract

Recovery from acute kidney injury can vary widely in patients and in animal models. Immunofluorescence staining can provide spatial information about heterogeneous injury responses, but often only a fraction of stained tissue is analyzed. Deep learning can expand analysis to larger areas and sample numbers by substituting for time-intensive manual or semi-automated quantification techniques. Here we report one approach to leverage deep learning tools to quantify heterogenous responses to kidney injury that can be deployed without specialized equipment or programming expertise. We first demonstrated that deep learning models generated from small training sets accurately identified a range of stains and structures with performance similar to that of trained human observers. We then showed this approach accurately tracks the evolution of folic acid induced kidney injury in mice and highlights spatially clustered tubules that fail to repair. We then demonstrated that this approach captures the variation in recovery across a robust sample of kidneys after ischemic injury. Finally, we showed markers of failed repair after ischemic injury were correlated both spatially within and between animals and that failed repair was inversely correlated with peritubular capillary density. Combined, we demonstrate the utility and versatility of our approach to capture spatially heterogenous responses to kidney injury.

## Introduction

Acute kidney injury (AKI) is a common clinical problem associated with significant mortality and cost^1^. Immunofluorescence (IF) imaging is a widely used technique to study AKI in human biopsies and in animal models. This technique has the unique ability to simultaneously capture both expression levels of multiple proteins and the spatial relationships between the cells and tissues expressing them.

Recently, transcriptomic analyses of single cells have revealed a wide range of cell states after kidney injury^2–5^, consistent with the observation that recovery from AKI can be inconsistent and heterogenous^6^. Understanding the spatial organization of cells is critical to understand the pathogenesis of AKI and recovery, but this information cannot be measured directly in single cell -omics approaches. While spatial transcriptomic techniques may address some of these shortcomings, these techniques are expensive and require highly specialized equipment and training. In contrast, IF staining of tissue sections is straightforward, can confirm cell-level (or even sub-cellular) changes in expression, can measure spatial relationships between cells, and is accessible to any laboratory.

There are relatively few techniques^7–9^ available to extract spatial data from stained kidney tissue that can study 10^4^ cells or more per sample, as is routinely achieved with single cell approaches. More commonly, IF analyses require time-consuming scoring or counting procedures or semi-automated thresholding approaches. Though entire tissue sections are stained, out of necessity, analysis is performed only for a small number of sub-samples called regions of interest (ROIs). Therefore, the overall area analyzed represents only a fraction of the available stained tissue. When the methodology to select ROIs is not rigorously defined and there is significant heterogeneity within samples, these procedures can bias sample measurements.

Furthermore, good animal models of AKI, such as murine ischemia–reperfusion, produce results with significant animal-to-animal variation, even in well-trained hands^10,11^. Human samples are even more heterogeneous^12,13^. To overcome this inherent variability, larger sample sizes are required to achieve appropriate statistical power. However, the number of IF-stained samples that can be analyzed is limited by the time and cost associated with ROI-based analysis, which scales linearly with the number of samples and targets of interest. Often practical limitations result in underpowered analyses. Techniques that can unlock the wealth of spatial data available in IF-stained tissue sections and that can be deployed across large sample sizes at low cost would significantly enhance the analysis of AKI.

In the last decade, there has been an explosion in the use of deep learning to analyze imaging data^14^. These tools can emulate manual approaches by performing image segmentation, which assigns pixels or regions of an image to classes (e.g. positive or negative staining). In theory, these approaches can overcome the time and cost limitations of ROI-based strategies by performing analysis computationally over areas impractical to analyze using manual or semi-automated techniques. Despite their potential, these approaches have received only limited attention for studying kidney disease^8,9,15–20^, in part because deploying these techniques across large numbers of samples in a cost-effective fashion is challenging.

In this report, we develop and describe a straightforward and accessible approach to apply these technologies to study kidney disease and build tools to extract and analyze the heterogeneity of AKI at both the tissue and cellular level for large areas and sample sizes.

## Results

### Overview

**Figure 1:**
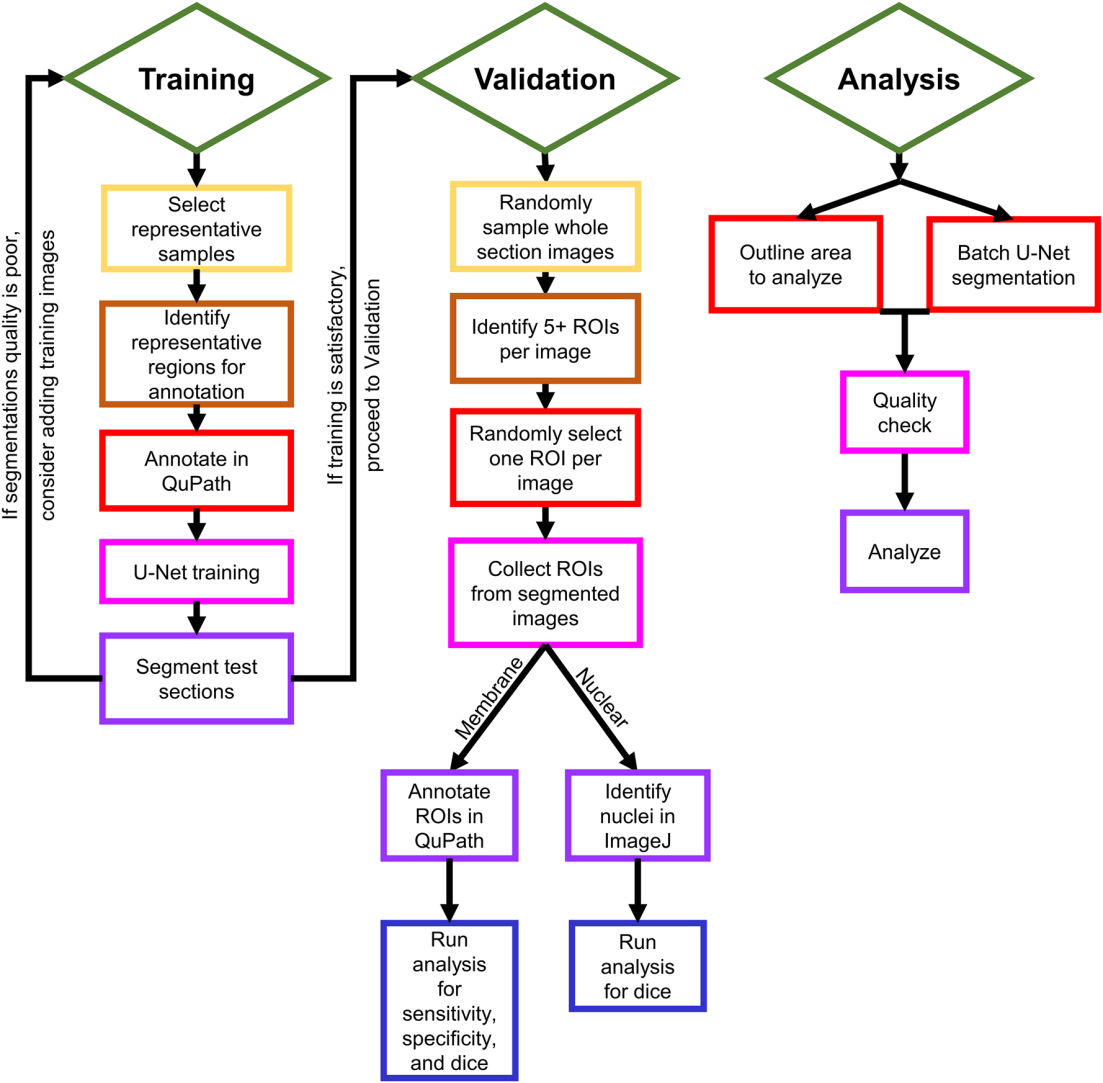
Flowchart of processing steps in each domain. Processing was divided into three domains: training, validation, and analysis. Each domain requires a library of whole-section, single-channel images as input. Detailed instructions for completing each flowchart task, including example data and example models, is available at https://github.com/Beamish-Lab/High-throughput-Image-Analysis-of-Kidney-Injury.

The pipeline we developed has three core domains: training, validation, and analysis, each composed of several modules (Fig. 1). Throughout our approach we utilized both QuPath^21^ and ImageJ^22^ to perform tasks required for processing raw image data for analysis, determined by the strengths of each platform. Detailed step-by-step instructions for each domain as well as ImageJ and QuPath macros that automate processing are available online (see “Methods”, “Code availability”). We selected the U-Net^23^ deep learning platform for deep learning-based segmentation because it is well-suited for analyses of IF images, but our approach can be performed with other segmentation tools^14^ compatible with batch processing.

### U-Net image segmentation of membrane selective stains

**Figure 2:**
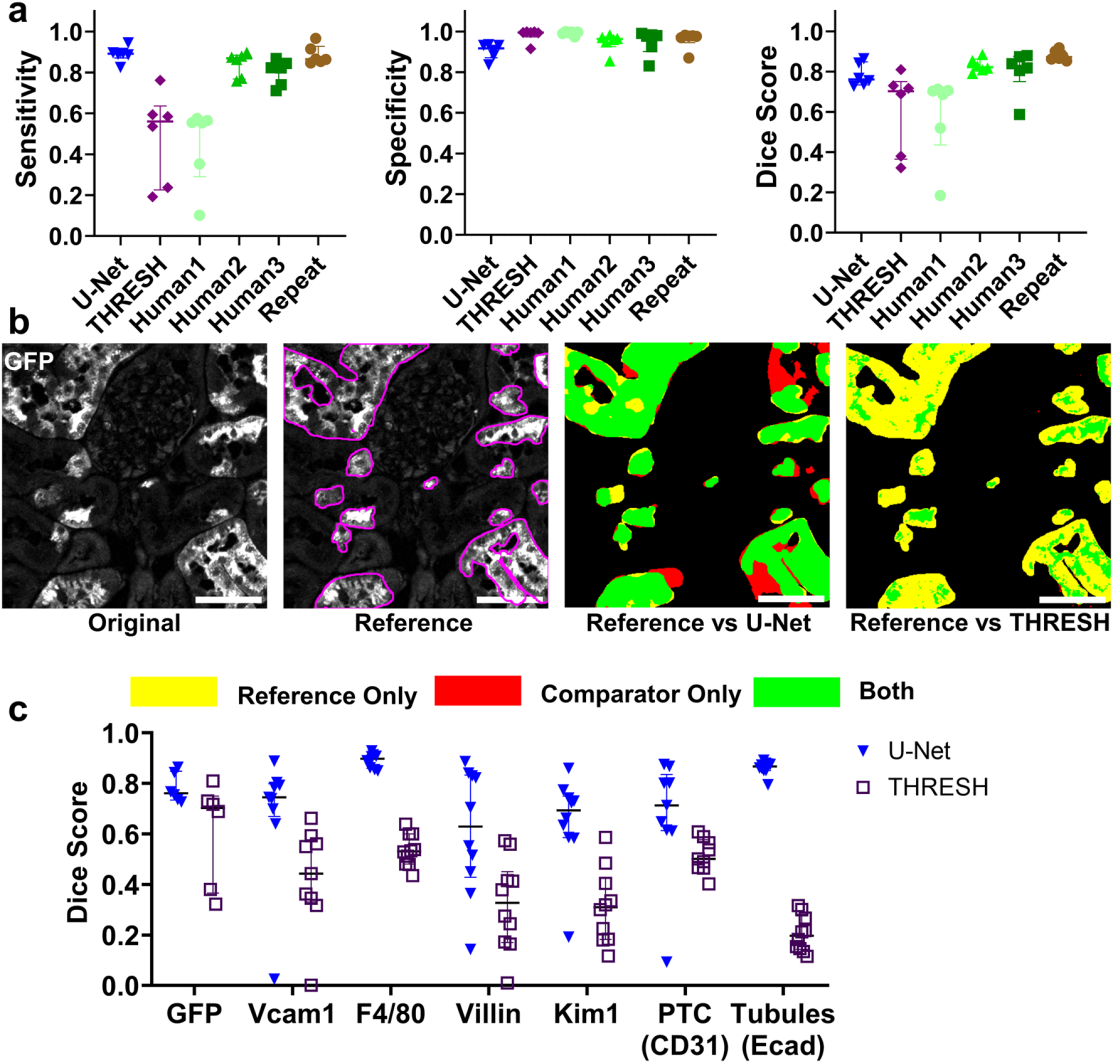
U-Net segmentation performance is similar to human annotation and is superior to thresholding for membrane-targeted stains. (**a**) U-Net model for identifying GFP positive proximal tubule cells was trained as described. Random sample ROIs were extracted from U-Net segmentations of whole kidney sections and compared with ROIs annotated by a reference expert observer to generate pixel-wise sensitivity, specificity and Dice score for each image. Median + interquartile range are shown. Performance was also compared with thresholding of a background subtracted section using ImageJ defaults (THRESH), three independent trained human annotators (Human1, Human2, Human3), and the reference observer’s re-annotation of blinded, randomly reordered, randomly rotated ROI images (Repeat). (**b**) Examples of one validation ROI image showing the original image, the outlines of the reference observer (magenta), and the comparison of the reference observer with U-Net segmentations and thresholding. Scale bar 50 μm. (**c**) U-Net models were trained for several additional membrane localized stains (Vcam1, F4/80, villin) as well as stain-defined structures Kim1 + tubules (Kim1), peritubular capillaries (PTC, marked by CD31), and tubules (marked by E-cadherin, Ecad). Performance was compared with thresholding.

One fundamental image analysis task is to identify areas or cells that express a protein target of interest. As a prototype, we obtained tissue sections from the kidneys of transgenic mice that express green fluorescent protein (GFP) in a subset of proximal tubule cells and stained them for GFP. From the resulting whole kidney images, we extracted eight 400 µm square ROIs, annotated the GFP positive cells, and used these images to train a U-Net model. To assess performance, we extracted an independent set of validation images from kidney sections not used for training. We compared U-Net segmentations, thresholding (using the default auto-threshold method for background subtracted whole sections), and three independent human observers to a reference observer. The reference observer also re-annotated randomly reordered and rotated versions of the reference images (Fig. 2a, “repeat”) after several weeks. We found that the trained U-Net model generated results with well-balanced sensitivity and specificity and an average Dice score of 0.78 (Fig. 2a). This result was similar to, or better than, other human observers and, remarkably, only slightly inferior to repeat annotation by the same observer (Dice score 0.88). Automated default thresholding resulted in a high specificity but low sensitivity. This result was expected given that GFP tends to stain strongly in the apical brush border of proximal tubule cells, so thresholding preferentially selects this part of the cell (Fig. 2a,b). The variability between human observers highlights the imprecision and user-dependence of manual annotations without a significant performance improvement over U-Net segmentation. Furthermore, U-Net segmentations are orders of magnitude more efficient. Manual annotation of one 400 × 400 µm image by a well-trained observer takes approximately 5 min. This manual rate translates to nearly 12 h for just one typical mouse kidney section compared with 2–4 min using an entry-level GPU-accelerated cloud computer to perform U-Net segmentation.

We next deployed this training approach for a variety of other membrane-bound protein targets relevant to AKI and kidney physiology including villin, Vcam1, and F4/80, which mark normal proximal tubule, failed repair proximal tubule, and macrophages, respectively (Fig. 2c, Supplementary Fig. S1). Though performance varied from target to target, the resulting performance was uniformly superior to default thresholding alone and had similar Dice scores to GFP (range: 0.61–0.89).

We next investigated the ability of U-Net segmentations to identify relevant higher-order structures in the kidney. First, we trained a model to identify injured tubules using Kim1 as a marker. Both segmentation and default thresholding had high specificity, but segmentation was more sensitive resulting in a Dice score of 0.65 vs 0.31 (Fig. 2c, Supplementary Fig. S1). Next, we developed a U-Net model to distinguish peritubular capillaries using CD31 staining, given that changes in peritubular capillaries play a critical role in injury and repair after AKI^24–26^. Remarkably, this model excluded glomerular capillary tufts with near-perfect accuracy (Supplementary Fig. S2). Even when assessed in ROIs that excluded glomeruli, the U-Net model clearly outperformed a default thresholding approach alone (Dice score 0.67 vs 0.51) though was limited by detection of the endothelium of larger vessels in addition to capillary structures (Fig. 2c, Supplementary Fig. S1, Supplementary Fig. S2). Last, we tested whether U-Net could distinguish tubular structures from the interstitium. We selected the membrane protein E-cadherin, which is used commonly to identify tubular epithelial cells^27^ and is expressed in a distinctive pattern that highlights cell–cell borders (Supplementary Fig. S1). Tubules with this pattern are easily recognized by human observers but not by thresholding alone, making this stain a good example to test the capabilities of U-Net to detect higher order structures. The U-Net model substantially outperformed default thresholding, as expected (Dice score 0.86 vs 0.20, Fig. 2c, Supplementary Fig. S1). When combined in the same sections, segmentations for peritubular capillaries and tubules segregated well such that capillaries were present almost exclusively in the interstitium (intersection over union 0.051 ± 0.007, Supplementary Fig. S3).

In some instances, ImageJ’s default thresholding performance was clearly suboptimal and may not be a well-matched benchmark for comparison. To better assess this, we analyzed the performance of all thresholding methods available in ImageJ on our validation data sets (Supplementary Fig. S4). Thresholding was performed either (1) over the entire kidney section first (as would be the case for analyzing whole kidney sections) followed by extraction of the output for each ROI or (2) for each ROI individually, approximating a local thresholding strategy. While some methods performed slightly better for selected stains, no thresholding method consistently outperformed U-Net across all stains tested. Likewise, local thresholding did not produce a consistent improvement in accuracy. Overall, these results highlight the superior generalizability of a U-Net approach to segmentation over thresholding methods.

In summary, U-Net models efficiently identified cells positive for cell surface markers and higher-order kidney structures with similar reproducibility as human observers.

### U-Net image segmentation of nuclear stains

**Figure 3:**
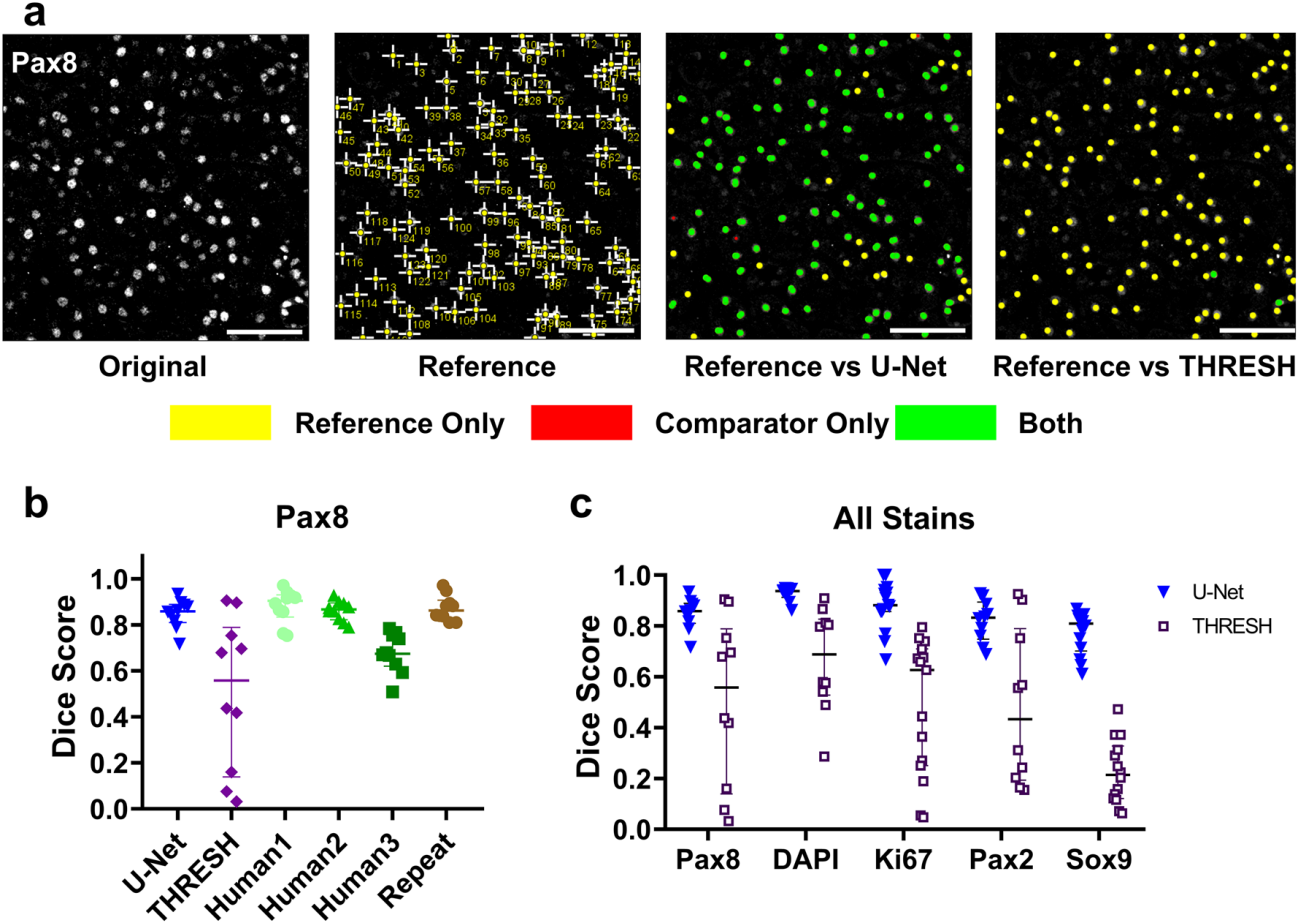
U-Net segmentation performance is similar to human annotation and superior to thresholding for nuclear-localized stains. (**a**) U-Net was trained to identify unambiguously positive Pax8 expressing nuclei as described. Random sample ROIs were extracted from U-Net segmentations of whole kidney sections and the coordinates of individual positive nuclei were determined. Nuclear coordinates were compared with the locations of nuclei marked by an expert reference annotator (crosses). Pairs of matching coordinates were determined (within 5 μm, only one match per annotation, marked by a pair of green dots linked by a green line). Scale bar = 50 μm. (**b**) Matched and unmatched coordinates were used to calculate a Dice score for each image comparing the reference observer to U-Net, thresholding of the background-subtracted section (THRESH), independent trained human annotators (Human1, Human2, Human3), and the reference observer’s re-annotation of blinded, randomly reordered, randomly rotated images (Repeat). Median + interquartile range are shown. (**c**) U-Net models were trained for several additional nuclear-localized stains (DAPI, Ki67, Pax2, and Sox9). Dice scores were compared with thresholding.

Because many targets of interest in AKI are expressed or active in the nucleus, we also explored how U-Net segmentation detected nuclear stains. As an example, we studied Pax8 which is expressed by epithelial cells to different degrees throughout the nephron^28^. Thus, Pax8 represents a stain with variable expression where thresholding can be ambiguous. We trained our U-Net model to detect clearly positive nuclei (Pax8 high) and ignore ambiguous nuclei. Because nuclei are clear and distinct, we quantified the results in terms of number of nuclei detected, rather than area fraction as with membrane stains above. As with membrane stains, we compared a reference observer with U-Net segmentation, thresholding, additional human observers, and repeated annotation by the reference observer (randomized and rotated) (Fig. 3a, b). We noted that performance of automated default thresholding depended on the relative background in each ROI and was generally inferior to segmentation (Fig. 3). As before, the performance of U-Net segmentation was similar to that of human observers. The data also highlight the inherent variability between human observers in an ambiguous identification task (Fig. 3b).

Next, we tested how U-Net segmentation compared with automated thresholding for DAPI staining and Ki67, which mark all nuclei and the nuclei of proliferating cells, respectively. Positive staining in these examples is less ambiguous. Because of the highly prototypical nature of DAPI, segmentation performed extremely well and clearly outperformed automated default thresholding (Dice score 0.93 vs 0.67, Fig. 3c, Supplementary Fig. S5). The most common segmentation error was a failure to separate closely adjacent or overlapping nuclei in our 2D images. Performance was also superior for Ki67 in injured kidney sections (Dice score 0.88 vs 0.48, Fig. 3c, Supplementary Fig. S5). We next tested staining for Pax2 which has variable expression (generally exclusive to the loop of Henle and the collecting ducts in uninjured kidneys) with similar results to Pax8 (Fig. 3c, Supplementary Fig. S5). Last, we tested the performance for nuclear Sox9, a marker involved in the early response to injury, using an antibody that clearly detects positive nuclei but also detected non-nuclear epitopes (see example Supplementary Fig. S5). Segmentation was able to distinguish positive nuclei clearly from background in injured kidney sections (Dice score 0.77 vs 0.23, Fig. 3c, Supplementary Fig. S5) and far outperformed thresholding approaches. As with membrane stains, we also compared U-Net segmentations to other thresholding methods in ImageJ and similarly found variable results without identifying any method with consistently better performance (Supplementary Fig. S6).

### Segmentation quantifies whole cortex response to folic acid injury

We next tested this strategy to analyze the response to injury across whole mouse kidney sections using folic acid nephrotoxicity. Animals analyzed developed severe kidney dysfunction by 2 days after injection and returned to baseline function 28 d after injury as measured by blood urea nitrogen (BUN) and serum creatinine (Supplementary Fig. S7). We also confirmed the expected pattern of injury and recovery by evaluation of hematoxylin and eosin-stained sections (Supplementary Fig. S7). This model generally has less variability than ischemia reperfusion^29^ with coefficients of variation for BUN at each timepoint ranging from 1.8 to 7.6% compared with 25–33% for BUN after ischemic injuries^10^ (Supplementary Fig. S7).

**Figure 4:**
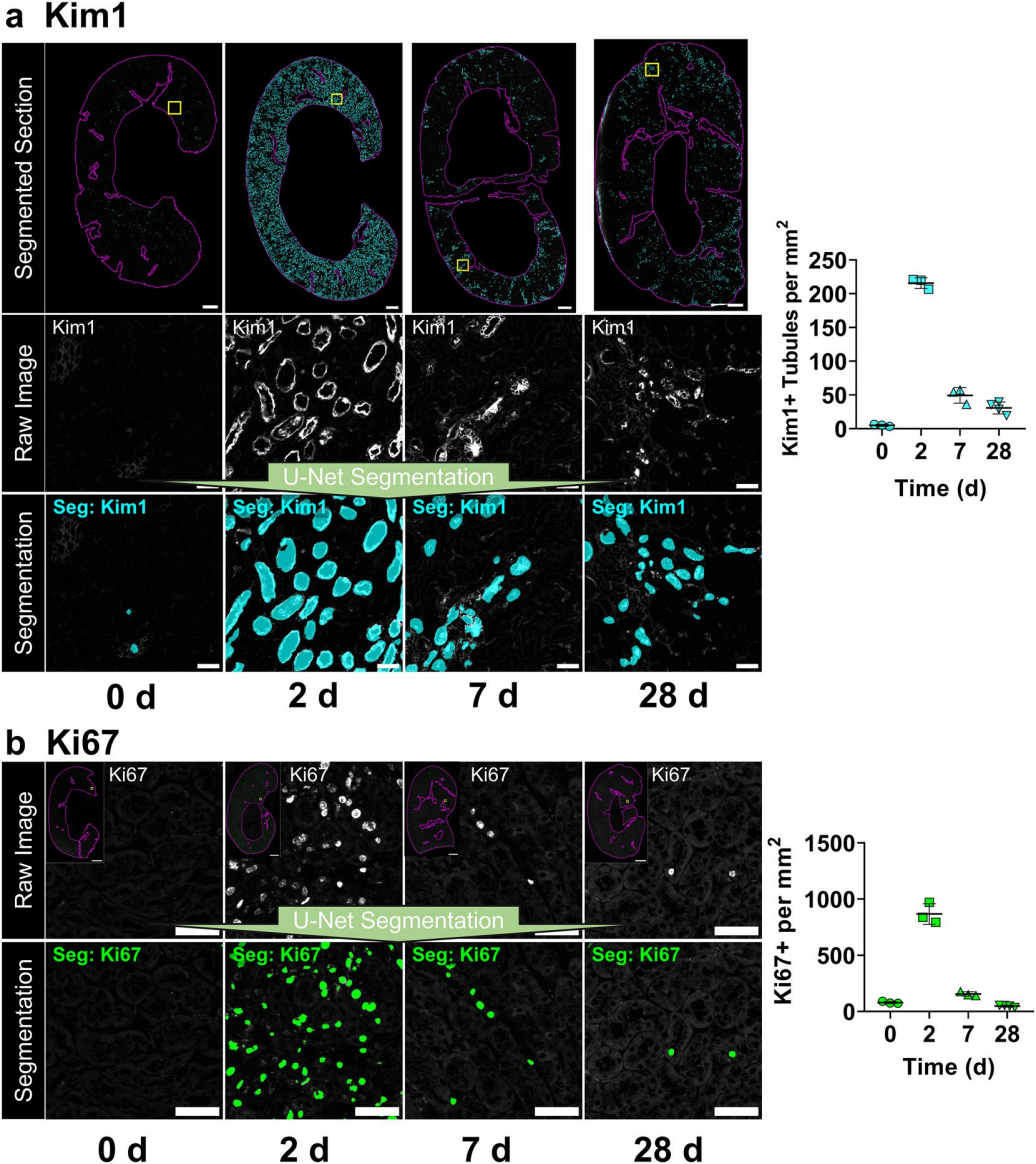
U-Net segmentation closely tracks the evolution of folic acid injury and identifies areas of chronic injury. (**a**) Analysis was performed over the entire cortex + OSOM (magenta outline) of coronal kidney sections obtained at various times after folic acid administration. Segmentation results were quantified as number of Kim1 + tubules per area. Representative regions (yellow box on whole-section images) are shown with the segmentation (Seg, cyan) result overlayed on the raw image (in grayscale). U-Net segmentation identified areas of persistent Kim1 expression representing areas of chronic injury 28 days after injury (28 days inset). (**b**) Overlay of Ki67 segmentation results, quantified as Ki67 + nuclei per area. For both analyses, 3–4 sections per time point were analyzed. Scale bars: 1 mm (whole kidney), 50 μm (insets).

We selected Kim1 + tubules and Ki67 as prototypical markers and performed analysis for the cortex and outer strip of the medulla (OSOM). First, we generated a montage of all the U-Net segmentations within the cortex + OSOM (N = 13 sections, Supplementary Fig. S8). This step confirms accurate, artifact-free segmentation and provides an overview of the results. Next, analysis of U-Net segmentations showed low Kim1 and Ki67 staining at baseline which rose to high levels at 2 days, then recovered over the next 4 weeks (Fig. 4), closely matching the creatinine and BUN (Supplementary Fig. S7). In contrast to the return to baseline of creatinine and BUN, our analysis detected a small population of tubules that continued to have some Kim1 expression 4 weeks after injury compared with baseline (30.7 ± 8.9 vs 5.0 ± 1.7 tubules per mm^2^, *p* = 0.013, ANOVA with Tukey’s post hoc test), indicating areas of failed repair (highlighted in Fig. 4 inset, 28 days) but that Ki67 had returned to near baseline levels (80.0 ± 9.8 vs 47.8 ± 8.7 nuclei per mm^2^, *p* = 0.79, ANOVA with Tukey’s post hoc test).

We also used this dataset to assess the reproducibility of U-Net segmentation results, by repeating staining on a serial section for 0, 2, and 28 d. Model performance on serial sections was tightly correlated for both Kim1 (slope 1.03, Pearson r = 1.00, *p* < 0.0001) and Ki67 (slope 1.56, Pearson r = 0.89, *p* < 0.0001, Supplementary Fig. S9), indicating highly reproducible performance.

We also compared U-Net segmentations with thresholding (Supplementary Fig. S10). We reanalyzed Ki67 and Kim1 images in the folic acid nephrotoxicity set using the “Triangle” and “Mean” threshold methods respectively because these methods showed the best performance in our validation cohorts (as shown in Supplementary Fig. S6 and Supplementary Fig. S4, respectively). For Ki67, though we found grossly similar trends, thresholding tended to estimate a larger population of proliferating cells. Upon inspection, these were false positive detections (Supplementary Fig. S10). Despite comparable performance in our validation cohort, thresholding for Kim1 grossly underperformed, especially in samples with low levels of Kim1 staining (Supplementary Fig. S10). These results highlight the advantages of U-Net segmentation over thresholding when generalizing to larger areas and samples with a diverse range of staining patterns.

### Analysis using alternative machine learning segmentation tools

There are many possible tools available to perform image segmentation. Like U-Net, ilastik^30^ has a user-accessible interface to facilitate training and batch processing. As a proof of principle, we trained the default Random Forest pixel classifier in ilastik to segment Ki67 positive nuclei using the same training set used to train the Ki67 U-Net segmentation model. We then assessed performance using a set of validation images as in Fig. 3. The performance of the ilastik classifier was only modestly inferior to U-Net (Dice score 0.74 vs 0.88 for U-Net, Supplementary Fig. S11). When deployed to analyze Ki67 positive cells after folic acid injury, as in Fig. 4, ilastik produced similar results compared with U-Net (Supplementary Fig. S11). However, we were unable to produce a robust ilastik pixel classifier for more complex structures such as Kim1 + tubules. Nonetheless, these results highlight the ability of our analysis pipeline to accommodate alternative segmentation platforms.

### Segmentation analysis is robust with lower resolution imaging

Images used for the above analysis were generated from scanned slides at a resolution of 0.5 µm/pixel. We also generated images of injured kidney sections (ischemia–reperfusion, 14 days) using a standard fluorescence microscope with a motorized stage (resolution 1.3 µm/pixel). Sections were analyzed using unmodified U-Net models. Despite a compromised image quality, U-Net segmentations performed well for Kim 1 (Dice score 0.71 vs 0.52 for automated default thresholding, Supplementary Fig. S12) and Ki67 (Dice score 0.79 vs 0.49 for automated default thresholding, Supplementary Fig. S12). These results highlight the generalizability of this approach for widely available microscopy setups.

### U-Net segmentations capture heterogeneous responses to ischemic AKI

**Figure 5:**
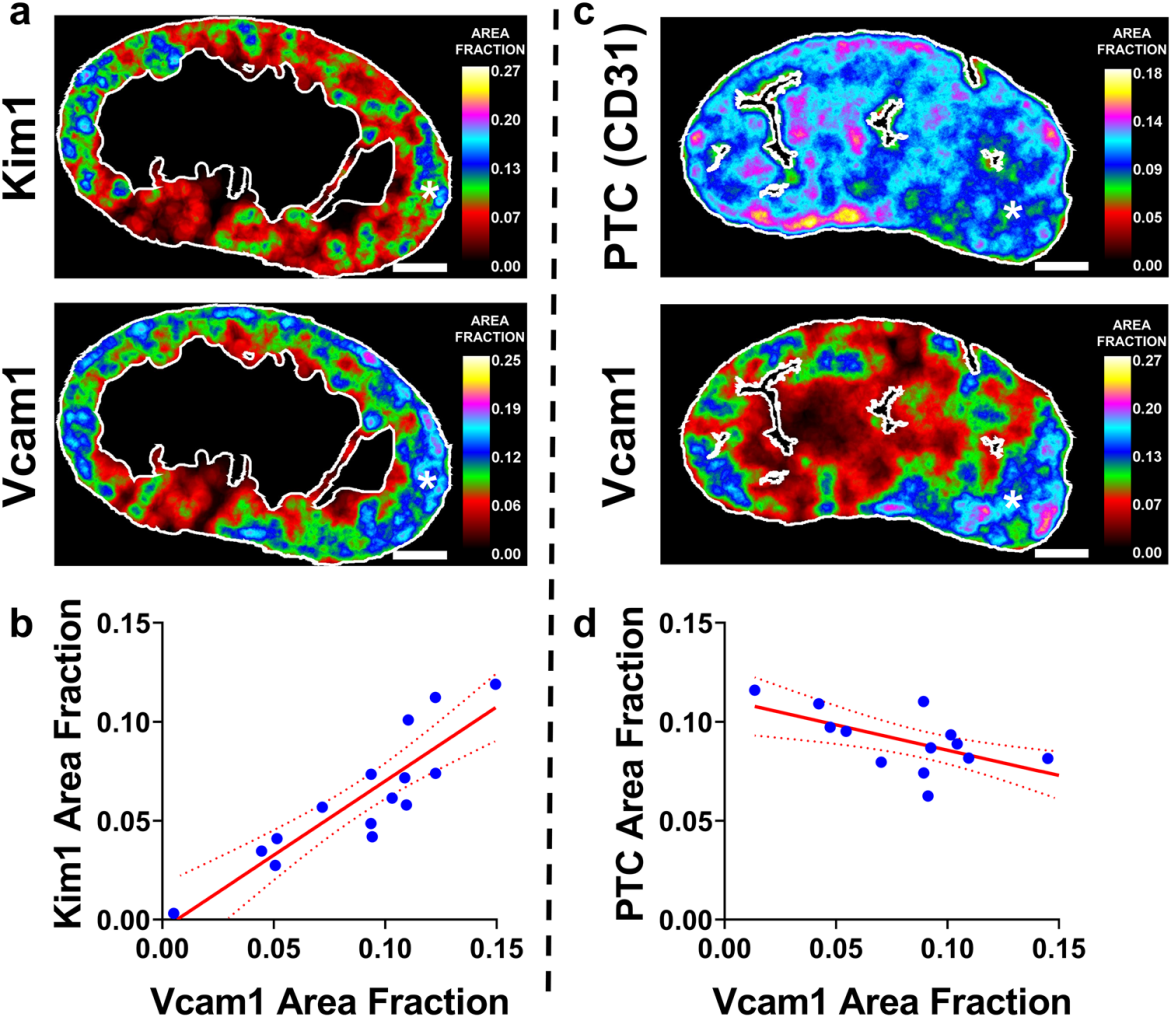
U-Net segmentations show a strong positive correlation between markers of failed repair and a negative correlation between failed repair and peritubular capillary density. The cortex and OSOM in coronal sections of injured kidneys were analyzed from mice sacrificed 14 d after ischemic kidney injury. (**a**) Kim1 and Vcam1 were co-stained and each channel was segmented using the U-Net models developed in Fig. 2. Heat maps of the segmentation area fraction of representative sections are shown (all sections are shown in Supplementary Fig. S15). Note areas with high Kim1 density correlate with high Vcam1 density (*). Scale bar = 1 mm. (**b**) The relationship between the segmentation area fractions of Vcam1 and Kim1 for each section is shown and demonstrates a tight correlation (Pearson r = 0.883, *p* < 0.0001). (**c**) Representative heatmaps for Vcam1 and peritubular capillaries (PTC) are shown (all sections are shown in Supplementary Fig. S16). Note that areas of Vcam1 staining tend to have lower PTC density (*). Scale bar = 1 mm. (**d**) The relationship between the segmentation area fractions of Vcam1 and PTCs for each section is shown and demonstrates a tight correlation (Pearson r = −0.67, *p* = 0.006). Dotted lines represent 95% confidence range for linear regression (**b,d**).

We next used U-Net segmentation to quantify the extent of failed repair, marked by Vcam1^2^, 14 days after severe unilateral ischemia, in a large cohort of animals (N = 15). Severe chronic injury was confirmed by review of hematoxylin and eosin-stained sections (Supplementary Fig. S13). Compared with the uninjured contralateral kidney, both Vcam1 and Kim1, averaged over the cortex + OSOM, were dramatically increased (*p* < 0.0001 for both, paired t-test, Supplementary Fig. S14), confirming the severity of injury. Because the contralateral kidney was preserved, BUN or creatinine were not expected to change substantially and were not measured. We next used the whole-section segmentations to generate heatmaps of Vcam1 and Kim1 density (as area fraction, averaged over a 200 µm radius) for each kidney (Fig. 5A, Supplementary Fig. S15). We observed substantial variability in recovery between animals (area fraction for Vcam1 0.005–0.150, coefficient of variation 42%) and substantial heterogeneity within sections from the same animal (Fig. 5a, Supplementary Fig. S15), as is typical with this model^10^. Though a marker of acute injury, Kim1 remains upregulated in areas of failed repair 14 d after injury^2^. We found that the average density of Kim1 and Vcam1 staining was very tightly correlated across animals (Pearson r = 0.88, *p* < 0.0001, Fig. 5b). To assess the spatial correlation of Kim1 and Vcam1 staining, we divided the cortex + OSOM of each section into non-overlapping close-packed 200 µm radius ROIs (Supplementary Fig. S15) and compared densities within each ROI (N = 8509 total). This analysis revealed a significant spatial correlation between Vcam1 and Kim1 (Pearson r = 0.68, *p* < 0.0001), as has been suggested previously^2^.

Since changes in peritubular capillary density likely contribute to failed recovery^24–26^, we conducted a second similar analysis comparing Vcam1 and peritubular capillary density (marked by CD31 as described above). Non-consecutive sections from the same animals that were used to assess Vcam1 and Kim1 above (N = 15) were processed and analyzed. Heatmaps of peritubular capillary density and Vcam1 were generated (Fig. 5c, Supplementary Fig. S16). These sections showed the same increase in Vcam1 density as above (Supplementary Fig. S14), and Vcam1 density was tightly correlated, by animal, with the densities measured above (Pearson r = 0.86, *p* < 0.0001, Supplementary Fig. S14), highlighting the reproducibility of our approach. We measured an 8.2% decrease in the peritubular capillary density in injured cortex + OSOM (*p* = 0.003, paired t-test, Supplementary Fig. S14). Peritubular capillary density was negatively correlated with Vcam1 density on a per animal basis (Pearson r = −0.67, *p* = 0.006, Fig. 5d). We also noticed that areas of high Vcam1 staining often associated with areas with low peritubular capillary density, especially in kidneys with the most heterogenous response. Indeed, when correlations were analyzed in non-overlapping 200 µm close-packed ROIs across all the available samples, we found a modest but statistically robust negative correlation (N = 9877, Pearson r = −0.23, *p* < 0.0001, Supplementary Fig. S16).

We also performed analysis of Vcam1 and PTC density using the thresholding methods that performed best for these stains (“Triangle” and “Li”, respectively). Using this approach, the ability to detect the negative correlation between Vcam1 and PTC density was lost owing to false positive detection of glomerular capillaries and debris in the injured kidney tissue, both spatially and between samples (Supplementary Fig. 17). This failure of thresholding methods highlights the unique capabilities of deep learning approaches for spatial analysis of complex structures after kidney injury.

## Discussion

Immunofluorescence (IF) imaging has been fundamental to the analysis of kidney disease for decades. However, approaches to extract large-scale spatial data from tissues processed with these techniques remain limited. Deep learning can unlock an unprecedented level of data from biomedical images including mapping the mouse brain vasculature^31^, quantifying metastatic burden in whole organs^32^, identifying the course of unmyelinated nerve axons^33^, and tracking kidney development^34^. Our goal was to develop an approach to apply these technologies to the study of kidney disease and build tools to analyze the heterogeneity of AKI at both the tissue and cellular level.

No single deep learning approach is suitable for all kidney image analysis. Kidney researchers require a diverse array of tools specialized for specific types of samples and questions. For example, periodic acid-Schiff (PAS) staining is used routinely to study kidney disease. Recently, U-Net-based architectures have been reported for the segmentation of tissue compartments in PAS-stained kidney sections^16,19,20^. These tools use a large set of training data to produce remarkably accurate results. Similar approaches have been useful for other routine histologic stains^35,36^, and tools to support segmentation of routine kidney histology are now accessible online^37^. Another generalized approach uses non-specific stains for F-actin^9^ or nuclear morphology^15^ which are correlated with validated cell-type markers to make inferences about cell identity using machine learning. Extensively trained models, such as these, are ideal for common, highly reproducible stains. Our approach complements these strategies. Focusing on single immunofluorescence-stained targets, chosen for their biologic relevance and high contrast, reduces the training set size needed, allowing our approach to be adapted efficiently for new targets as scientific questions evolve.

We provide tools to calculate and visualize metrics of segmentation accuracy which can be reported along with output measurements^38^. Our U-Net models were generated from small training sets but still showed performance was roughly equivalent to the variability between human observers, with Dice score generally ~ 0.6 to 0.8. These results agree with other reports which show considerable heterogeneity among human experts, even in simple tasks such as identifying positive nuclear staining^39^. We demonstrated that this level of performance is sufficient to extract meaningful biological data. Furthermore, U-Net segmentation was superior to optimized thresholding approaches, which did not generalize well and cannot be improved by further training. Improving U-Net model accuracy further requires expanding the library of training images or specialized deep learning frameworks, which are time-consuming to generate, risk overfitting, and are difficult to adapt if staining targets change. Furthermore, the variability between human observers highlights the challenges of defining “positive” and “negative” staining at the pixel level. In such a situation, a deep learning strategy has the advantage of consistently segmenting pixels ambiguous to the human observer.

Automating pre- and post-processing steps is essential to efficiently deploy our strategy for many samples. We used ImageJ as the hub for our analysis and developed ImageJ macros to automate processing. The ImageJ macro language is simple, well-documented, tailored specifically for images, and is familiar to many users with limited coding experience. Each code is self-contained and written to prioritize clarity over execution speed to make it easier for users to interpret and adapt. Furthermore, our approach was developed in a modular fashion. We have provided detailed step-by-step instructions (via a GitHub website; see “Methods”, “Code availability”) to guide users through each module. Experimental questions will naturally evolve as will techniques for training and analyzing biomedical data. Our modular approach allows users to exchange new algorithms or analyses optimized for new experimental questions. While weight files for our U-Net models are available via GitHub, additional fine-tuning should be anticipated to adapt each for new stains, sample processing techniques, and scientific questions.

Central to our approach is the U-Net deep learning platform^23^. There are many alternative machine and deep learning alternatives for segmentation^14^. However, the U-Net architecture has been used widely for a variety of applications^16,40–45^, as a de facto standard because it is well suited for analysis of grayscale biomedical images with features such as cells and nuclei. U-Net has well-documented instructions, can be implemented in ImageJ, and is easily trainable. U-Net requires GPU acceleration to operate efficiently, but its ImageJ plugin was designed to use remote servers to perform these calculations, which are inexpensive and readily available. U-Net recently has been deployed in the kidney for specific applications such as defining the spatial context for transcripts detected by in situ hybridization^17^ and performing podocyte morphometrics in human biopsy samples^18^. These examples reinforce the utility of U-Net for the more generalized analysis of immunofluorescence-stained kidney samples outlined here. Nonetheless, any segmentation approach can be substituted for this step of the analysis. We found that less-computationally demanding machine learning approaches, such as Random Trees or k-nearest neighbors^21,30^, could be substituted for highly stereotyped stains such as Ki67. Like with thresholding methods, in practice we were unable to obtain satisfactory results for more complex structures, suggesting that using a deep learning platform such as U-Net has the advantage of being generalized more easily to a range of segmentation tasks.

One key goal for our approach is to facilitate the analysis of large sample sizes. Mouse ischemia–reperfusion is an experimental model that replicates features of acute tubular necrosis, the most common cause of clinically severe AKI. Even in trained hands, this model results in a coefficient of variation typically between 25 and 33% for BUN^10^, which implies a minimum sample size of 9–18 animals (effect size 33%, alpha = 0.05, power = 0.80). We demonstrate tissue level response can show even greater variation (Supplementary Figs. S14, S15, S16). As a result, many analyses are underpowered both in terms of the regions analyzed for each animal and in the number of biological replicates (i.e. animals) assessed. Our approach analyzes tissue areas and sample numbers that are orders of magnitude larger than ROI-based approaches. This additional data allows us to capture variation within and between animals that otherwise would be difficult to measure. Here we quantified heterogeneous responses to folic acid and ischemic injury in mice, analyzed in the entire cortex + OSOM of tissue sections from many animals. In ischemic AKI, we showed that Vcam1 and Kim1 were spatially correlated, as has been reported^2^, but also that Vcam1 was correlated spatially with decreased peritubular capillary density, further supporting the important role of microvascular rarefaction in the recovery of kidney injury^46^.

Our approach has several key limitations. First, marker choice is critical for the success of our approach. We intentionally limited our study to high contrast stains amenable to manual annotation. While this encompasses many analyses relevant to studying AKI, stains with low contrast or ambiguous patterns of staining will not perform well in our pipeline. Second, acquiring images of complete sections can be time-consuming if automated scanning microscopes are not available, though as we demonstrate analysis can be performed accurately on images with lower resolution. Third, there is a significant time investment to learn, build, and deploy these pipelines. We have provided detailed step-by-step instructions (available at our GitHub website) to simplify and expedite this process. While each new stain will require manual annotation for training and validation, we have provided tools to facilitate generating this data and several pre-trained models that can be used directly or adapted. Fourth, some basic knowledge of programming is required to work with the macros included. Last, careful organization and file name conventions are required to store data and usher it through the analysis pipeline and as a result, small errors can break the chain of analysis.

In summary, we have presented a series of tools to enable the use of deep learning image analysis techniques to be trained, validated, and applied to the analysis of immunofluorescence images across large tissue areas and arbitrarily large numbers of samples. The accuracy of our approach is similar to human observers but captures orders of magnitude greater levels of spatial data from kidney sections without expensive or highly specialized equipment. The output from these analyses quantifies the heterogeneity within and between kidneys in response to kidney injury and can be applied to study a wide range of scientific questions.

## Methods

### Animals

All studies involving mice were approved by the Institutional Animal Care & Use Committee (IACUC) at the University of Michigan and performed in accordance with the NIH Guide for the Care and Use of Laboratory Animals and in accordance with ARRIVE guidelines. Studies included wild-type C57BL/6 mice or transgenic mice bred on the C57BL/6 background that carry phosphoenol pyruvate carboxykinase (PEPCK) Cre^47^ and a Cre reporter Ct(Rosa)26Sor^tm4(ACTB-tdTomato,-EGFP)luo^ which stably expresses membrane-bound enhanced green fluorescent protein (GFP) in cells that have expressed Cre as previously described^29^. PEPCK Cre is expressed in all three segments of the proximal tubule. Only male mice were used for these studies.

### Folic acid injury

Age-matched male mice were injected intraperitoneally with a single dose of 250 mg/kg folic acid in a total volume of 0.5 ml of 0.15 M sodium bicarbonate as previously described^29^. Mice were euthanized using carbon dioxide at 0, 2, 7, and 28 days after injection. Animals were immediately perfused with ice-cold phosphate buffered saline (PBS). Kidneys were explanted, divided through the hilum to generate two symmetric pieces, and fixed overnight in 4% paraformaldehyde/PBS.

### Unilateral ischemia–reperfusion injury

Age-matched male mice were anesthetized with isoflurane. The retroperitoneum was accessed by a small incision on the left flank. The kidney was gently retracted and fat was dissected from the pedicle. The renal artery and vein were clamped with a Schwartz clip (Roboz Surgical, RS-5459). After 30.0 min, the clamp was removed and reperfusion of the kidney was confirmed. The incisions were closed in layers. Body temperature was maintained at 38 °C throughout the procedure using an infrared homeothermic control system (Kent Scientific, RightTemp Jr). The contralateral kidney was not manipulated. Analgesia with carprofen (5 µg/g per 24 h) was provided for 48 h post-operatively (2 doses). Animals were euthanized 14 days after surgery. Samples were collected as described for folic acid injury above.

### Histology

Multiple samples were embedded in each paraffin block. Sections (~ 5 µm thickness) from 1 to 4 blocks were mounted per slide. Each slide, therefore, contained samples from several animals. Block location, orientation, and section shape were used to track and confirm the identity of each sample on the slide.

### Staining

Sections were deparaffinized and rehydrated using a graded series of xylene–ethanol–water solutions. Slides were treated with a citrate-based antigen unmasking solution (Vector Laboratories) in a pressure cooker for 10 min. Samples were then permeabilized with 0.5% Triton-X 100 in PBS, blocked with 20 mg/ml bovine serum albumin, and incubated overnight with primary antibodies (see supplemental table) in 0.1% Tween-20 in PBS (PBS-T). Sections were then extensively rinsed with PBS-T and incubated with species-specific fluorophore-conjugated secondary antibodies in PBS-T for 2 h at room temperature, rinsed with PBS-T, then PBS, then water, and quickly dried. Hydrophobic pen (which interferes with automated slide scanning) was removed by immersing the slides in xylene for 60 s. Xylene was then quickly evaporated under vacuum and the slides rehydrated in PBS. Samples were then treated with the TrueVIEW autofluorescence quenching kit (Vector labs) per manufacturer instructions. The final rinse before mounting included 1 µg/ml of DAPI. Samples were mounted with Prolong Diamond mounting medium (Invitrogen), allowed to cure overnight at room temperature protected from light, and stored at 4 °C protected from light until scanning.

### Imaging

Kidney sections were scanned at a resolution of 0.5 µm per pixel using a Vectra Polaris whole slide scanner (Akoya). For each project, exposure and gain were optimized for each channel and were kept constant for all slides. QuPath (version 0.3.1) was used to identify and extract each section from the whole slide image (which was a 2–8 GB *.qptiff file). Each section was then divided into separate single-channel images in *.tif format. The file name of these images was encoded with six levels of metadata needed for subsequent processing (in our studies: 1—genotype code, 2—GFP Cre reporter status, 3—sex, 4—ear tag, 5—experimental condition, and 6—stain; for example “WT_GFP_M_0001_IRI14_Kim1.tif”). The “Subtract Background…” feature of ImageJ was used to reduce background over each section (rolling ball radius = 50 pixels) prior to any downstream processing. For some sections, images were acquired with a Zeiss Apotome microscope (in standard epifluorescence mode) equipped with a motorized stage at a resolution of 1.3 µm per pixel. Images were acquired with 10% overlap and stitched together with the Bio-Formats plugin in ImageJ. Each section was imaged separately, with fixed acquisition parameters across the entire project, and processed to produce single-channel image files as above.

### Training

Training and segmentation were performed using the U-Net plugin in ImageJ (version 1.53t with Java 1.8.0_172, Fiji distribution) with segmentations processed remotely using an Amazon Web Services g4dn.xlarge EC2 instance running the Deep Learning Base AMI (Ubuntu 18.04 Version 44) with the caffe_unet_package_18.04_gpu_cuda10_cudnn7 U-Net package. 5–12 cortical ROIs were extracted for training annotation from multiple kidney sections and experimental conditions from the cortex + outer strip of medulla (OSOM). ROI size and number were determined to include sufficient positive detections that could be annotated by an expert observer in ~ 15 min, generally 200–1000 pixels square. ROIs that had obvious processing artifacts were excluded, such as blurring, scratches, or tissue folds. QuPath was used to annotate the images and the results were formatted for U-Net training (annotations were converted to an overlay with annotation class names in ImageJ). Some areas were designated “ignore” if annotation was ambiguous. From this training set, ~ 15% of the images were assigned for in-training validation (distinct from formal validation described below) to monitor training progress. The U-Net “Finetune Model” plugin feature was used to build new models. We used the U-Net example “2d_cell_net_v0.caffemodel.h5”^23^ as a starting point. Subsequent models were tuned from prior models with similar features (i.e. membrane or nuclear stains). Training was continued until the slope of the loss function approached zero, generally 5000 iterations for the first families of models derived from the U-Net example and 1000–2000 iterations for subsequent models for similar stains. For complex structures (peritubular capillaries, tubules), a second round of annotations was generated from preliminary predictions to convert the annotation task to a prediction correction task as described^16^, increasing the number of training images available for these more complex segmentations. Model weight and definition files are available via the GitHub website (see “Code availability”, below).

### Validation

Samples used for validation were strictly independent from training data sets. Within each project of identically stained slides, 10–12 sections that contained positive staining for the target of interest were randomly selected for inclusion using a random number generator. On each section, using the original image as a reference without knowledge of segmentation results, at least 5 ROIs were extracted from the cortex + OSOM (generally 400 × 400 pixels for membrane stains or 200 × 200 pixels for nuclear stains). ROI placement was random except that images with obvious artifacts were excluded. Of these, one ROI was randomly selected for inclusion in the final validation data set using a random number generator. This training set was annotated and used as a reference “gold standard” for subsequent analysis. Reference annotations were generated by a trained clinical nephrologist directly or generated by lab subordinates then reviewed, revised, and approved by the supervising nephrologist prior to analysis.

For membrane stains and structures, annotations were performed in QuPath. Because testing metrics become unstable as the positive fraction approaches 0 or 1, (i.e. overall performance depends only on a few pixels) only images with a positive area fraction of 0.1–0.9 were included. U-Net segmentation results for each ROI were extracted from the segmentation result of the entire section rather than segmenting the validation images separately. Thresholding results were generated for the entire background-subtracted kidney section using the various settings of the ImageJ “Threshold…” feature and extracted for each ROI as for segmentations. In some cases, thresholding was performed on ROIs individually. Unless specified otherwise, thresholding results shown were generated with the “default” method. For selected examples, the validation set was also provided to additional trained observers for annotation to assess inter-observer variability. The reference observer also re-annotated a randomly rotated and reordered validation set after at least 2 weeks to assess intra-observer variation. For membrane stains and structures, we used the binary annotation or segmentation result to calculate a sensitivity, specificity, and Dice score (equivalent to the F1 score for binary image segmentations, calculated as 2 × TP/[(TP + FP) + (TP + FN)], where TP is the number of true positive pixels, FP is the number of false positive pixels, and FN is the number of false negative pixels for the comparator relative to the reference) for each validation image. The closer the Dice score is to 1, the better the agreement between two binary images.

For nuclear stains, the analysis was adjusted from a segmentation task to a detection task, since generally the count of discrete positive nuclei is more relevant than the area of nuclei detected. For manual annotations, the centroid of each positive nucleus was marked with the “Multipoint” tool in ImageJ. Because testing metrics become unstable for only a small number of detections, only images with at least five positive nuclei, as determined by the reference observer, were included. For segmentations or thresholding, the centroid of each nucleus was determined using the “Fill Holes”, then “Watershed” and then “Analyze particles…” features of ImageJ (minimum particle area 9 μm^2^). The resulting pairs of coordinates were compared by matching each point in the reference sample with the closest point in the comparator set within 5 μm. Each point was matched only once. The resulting sets of matched and unmatched points were used to calculate a Dice score. Inter- and intra-observer variation was measured as with membrane stains. Detailed instructions and ImageJ code for performing these tasks are included on the GitHub website (see “Code availability” below).

### Sample analysis

Whole-section, background-subtracted, single-channel images were used as input. Prior to downstream steps, all sections were organized into a single montage for each group of animals. These images were reviewed for artifacts such as blurring, grossly irregular staining, scratches, or tissue folds. Images that failed this quality control step were reimaged or excluded from downstream analysis. An outline of the kidney section was generated in QuPath using a down-sampled image to speed processing time. The boundaries of the section were determined and large vessels, calyces, and obvious artifacts were excluded. Next, the edge of the OSOM was marked either using PEPCK Cre activity (marked by GFP) or based on morphological characteristics and combined with the whole-section outline to generate an outline of the cortex + OSOM. In parallel, original images were segmented with the appropriate U-Net model using a macro to automate batch processing.

The segmentation outputs were analyzed only in the cortex + OSOM area marked above. A montage of segmentations and section outlines was generated to identify any artifacts in the processing prior to analysis. For each section, measurements of detections per area, area fraction, and other metrics were calculated using the included analysis macro.

To visualize staining correlations, heatmaps of staining density were generated using the “Mean…” filter in ImageJ with a radius of 200 µm. Average staining density for each stain was correlated over the entire cortex + OSOM. Local spatial correlations were also analyzed by filling each cortex + OSOM outline with non-overlapping close-packed circles of 200 µm radius and then measuring the segmentation density in each pair of corresponding circles.

### ilastik

Selected images were analyzed with ilastik^30^ (version 1.4.0). The default Random Forest pixel classifier was trained using the same images used for training U-Net. Model features were determined using the “Filter” method. Training images were then annotated as specified in the software instructions. All areas marked as positive for U-Net training also were marked as positive within ilastik. Areas not annotated for U-Net training were marked as negative in ilastik. After training, full-size images, identical to those processed using U-Net, were segmented using the “Batch Processing” feature in ilastik with the “Simple Segmentation” export setting. The resulting segmentations were used for validation or downstream analysis exactly as performed with U-Net.

## Supplementary Information

Supplementary Information.

## Data Availability

The full-size images and datasets used in the current study are available from the corresponding author on reasonable request.

## Code availability

All code used in this manuscript (Fiji/ImageJ and QuPath macros), detailed instructions for use, and example images are publicly available at https://github.com/Beamish-Lab/High-throughput-Image-Analysis-of-Kidney-Injury.
