# Supplementary material for: High-throughput image analysis with deep learning captures heterogeneity and spatial relationships after kidney injury
Source: Sci Rep. 2023 Apr 19;13:6361. doi: 10.1038/s41598-023-33433-3 (PMC10115810; doi:10.1038/s41598-023-33433-3)
Supplement: Supplementary file 1 — Supplementary Information. [file 41598_2023_33433_MOESM1_ESM.pdf]

SUPPLEMENTARY FIGURES AND TABLES

High-throughput image analysis with deep learning captures heterogeneity and spatial relationships after kidney injury

Madison C. McElliott[1], Anas Al-Suraimi[1], Asha C. Telang[1], Jenna T. Ference-Salo[1], Mahboob Chowdhury[1], Abdul Soofi[2], Gregory R. Dressler[2], Jeffrey A. Beamish[1,*]

[1]Department of Internal Medicine, Division of Nephrology, [2]Department of Pathology, University of Michigan, Ann Arbor, Michigan.

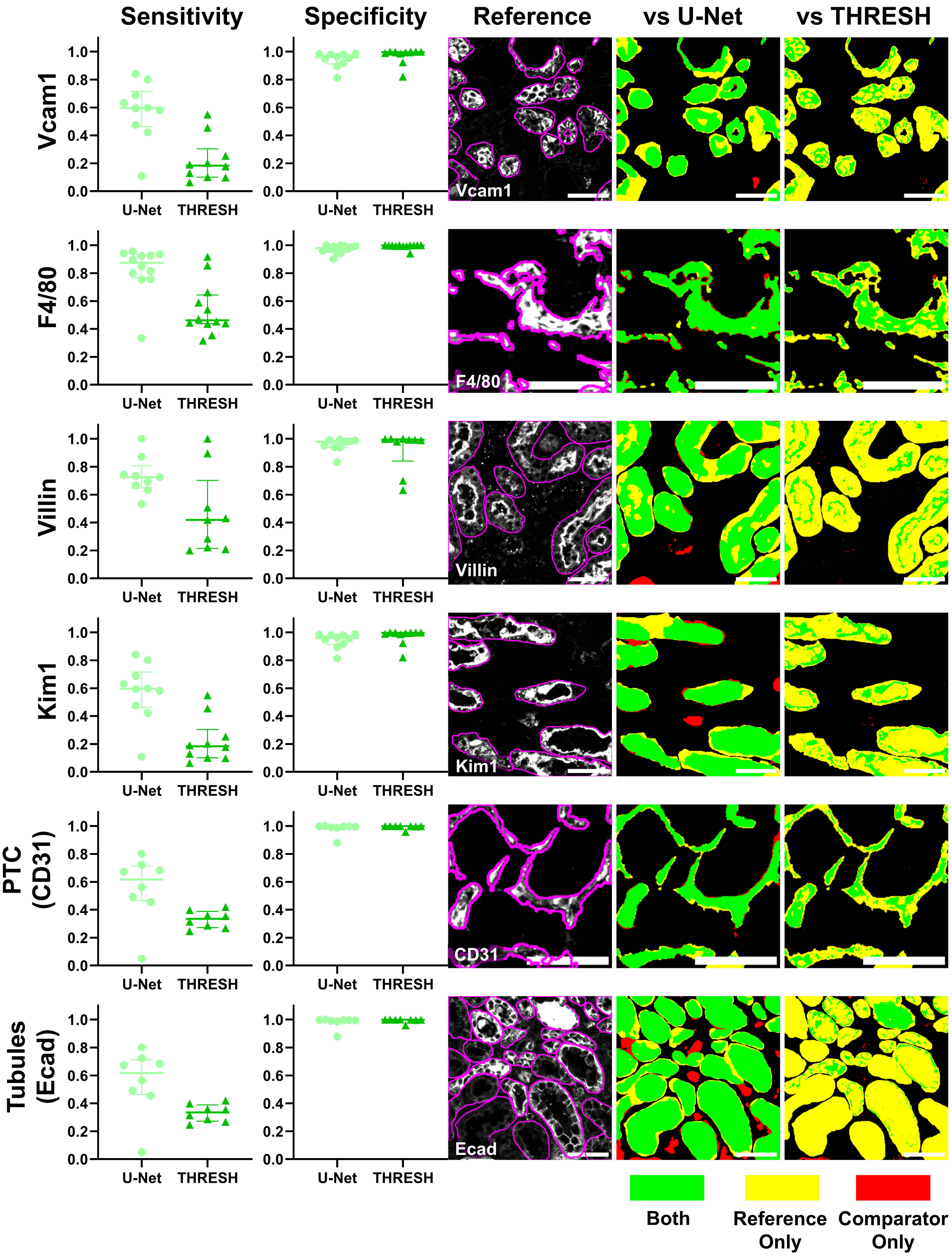

Supplementary Figure S1. Sensitivity, specificity, and representative images of U-Net segmentations and thresholding (THRESH) for stains and structures shown in Fig. 2: Vcam1, F4/80, villin, Kim1+ tubules (Kim1), peritubular capillaries (PTC, marked by CD31), and tubules (marked with E-cadherin, Ecad). Median and interquartile range are shown for sensitivity and specificity calculations. Annotations by the reference observer are shown as magenta outlines in the “reference” column of images. Scale bar = 50 μm.

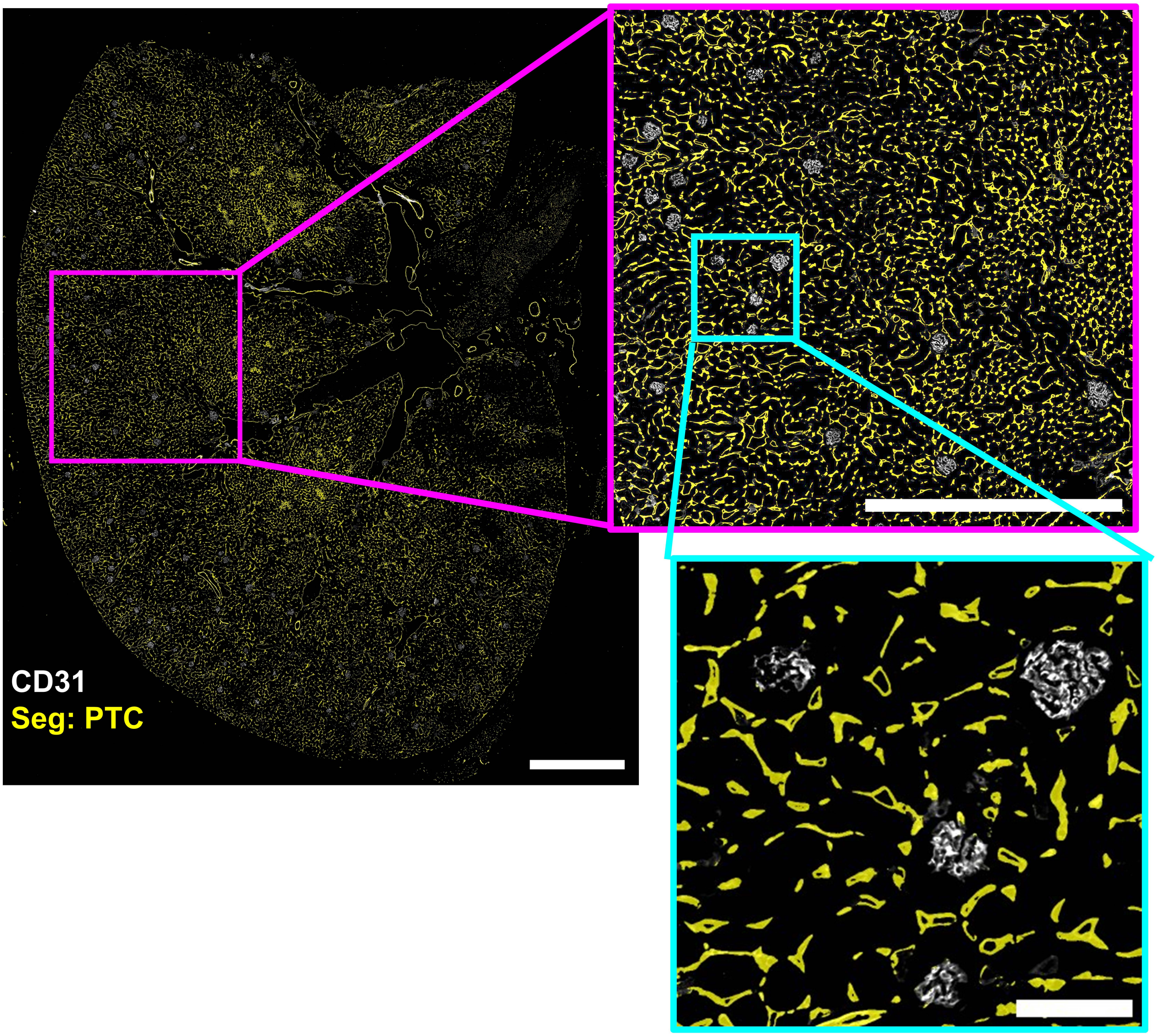

Supplementary Figure S2. Peritubular capillary segmentations exclude glomeruli. A U-Net model was trained to segment CD31 staining for peritubular capillaries (PTC) and exclude glomerular capillaries. Representative whole kidney sections with CD31 staining are shown with a yellow overlay of the U-Net PTC segmentation model output. Scale bars: 1 mm (full image), 1 mm (intermediate ROI), 100 µm (small ROI).

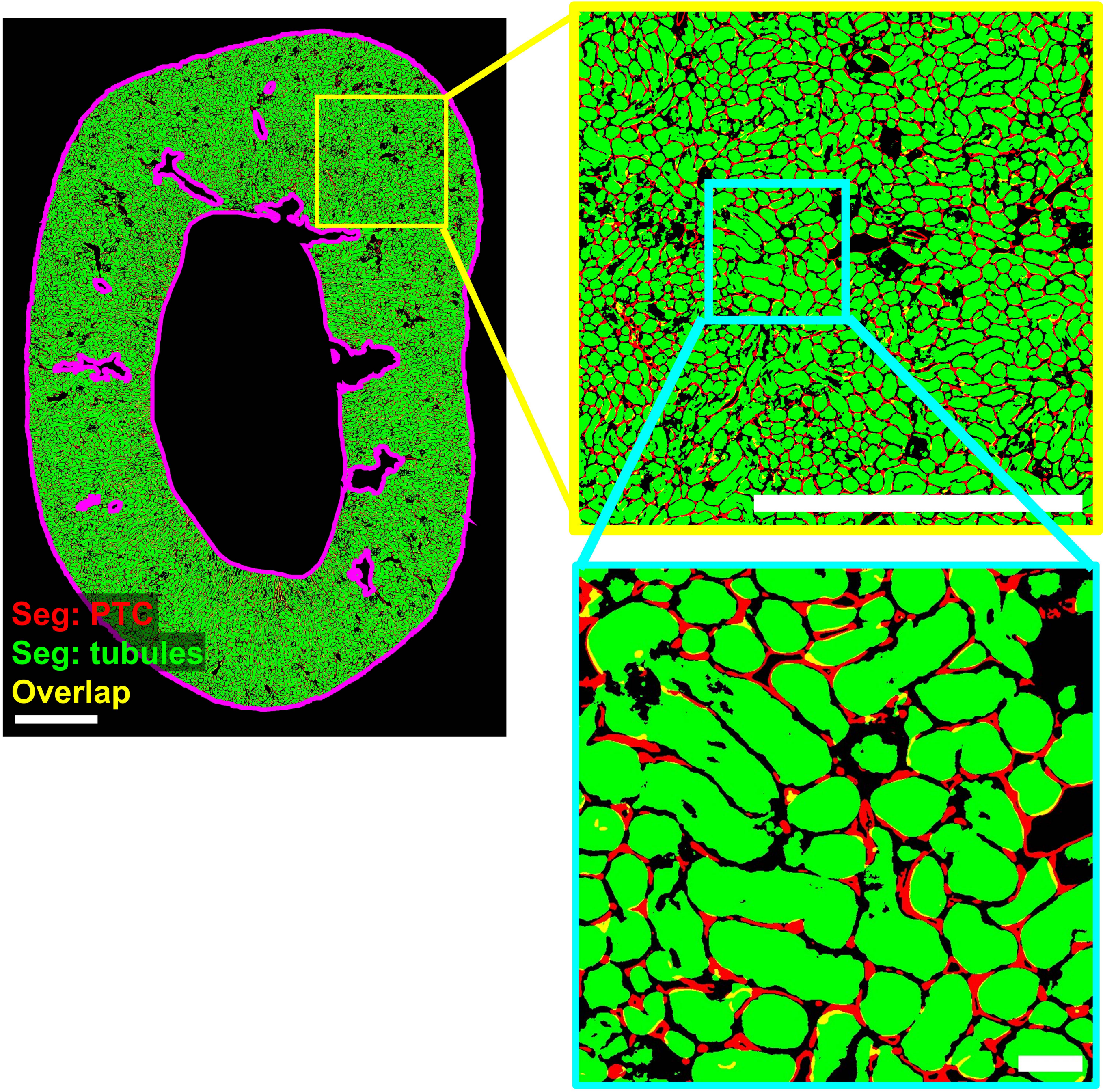

Supplementary Figure S3. Segmentations for peritubular capillaries (marked by CD31) were localized to the interstitial spaces between tubules confirming the accurate localization of these higher order structures. The degree of overlap was assessed by calculating an intersection over union (IoU) for 15 independent sections in the cortex + OSOM (IoU = 0.051 ± 0.007, mean ± std dev). Scale bars: 1 mm (full image), 1 mm (intermediate ROI), 50 µm (small ROI).

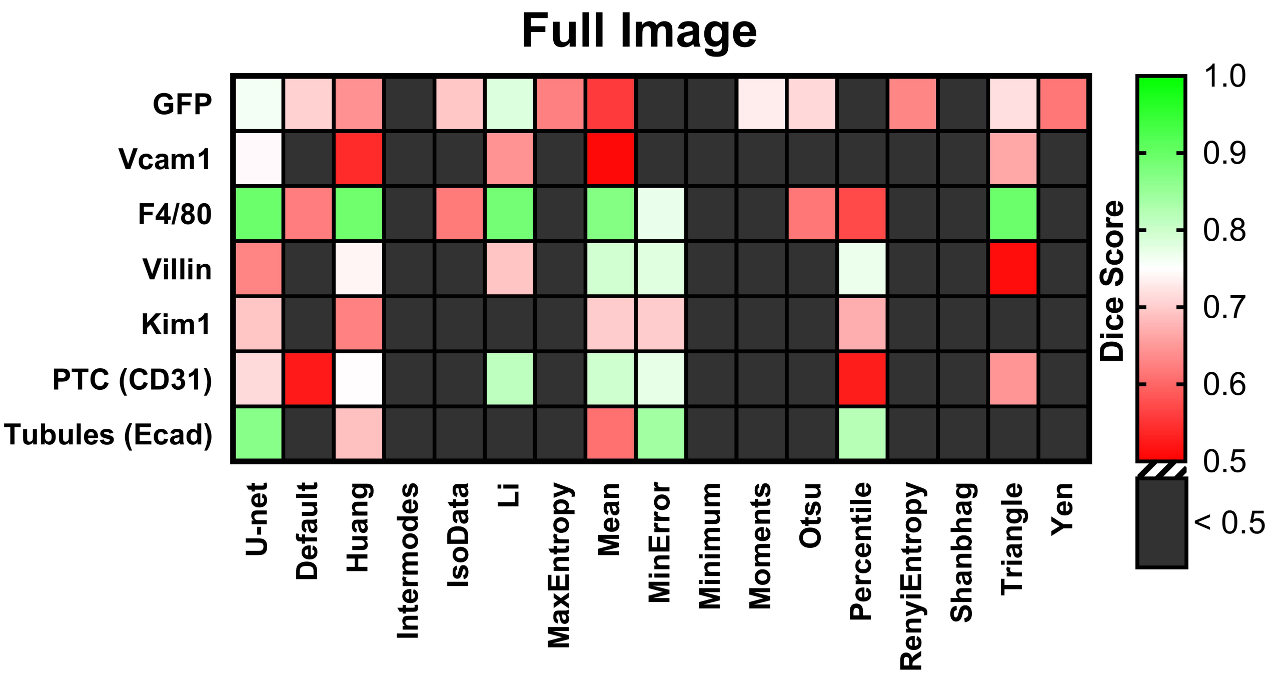

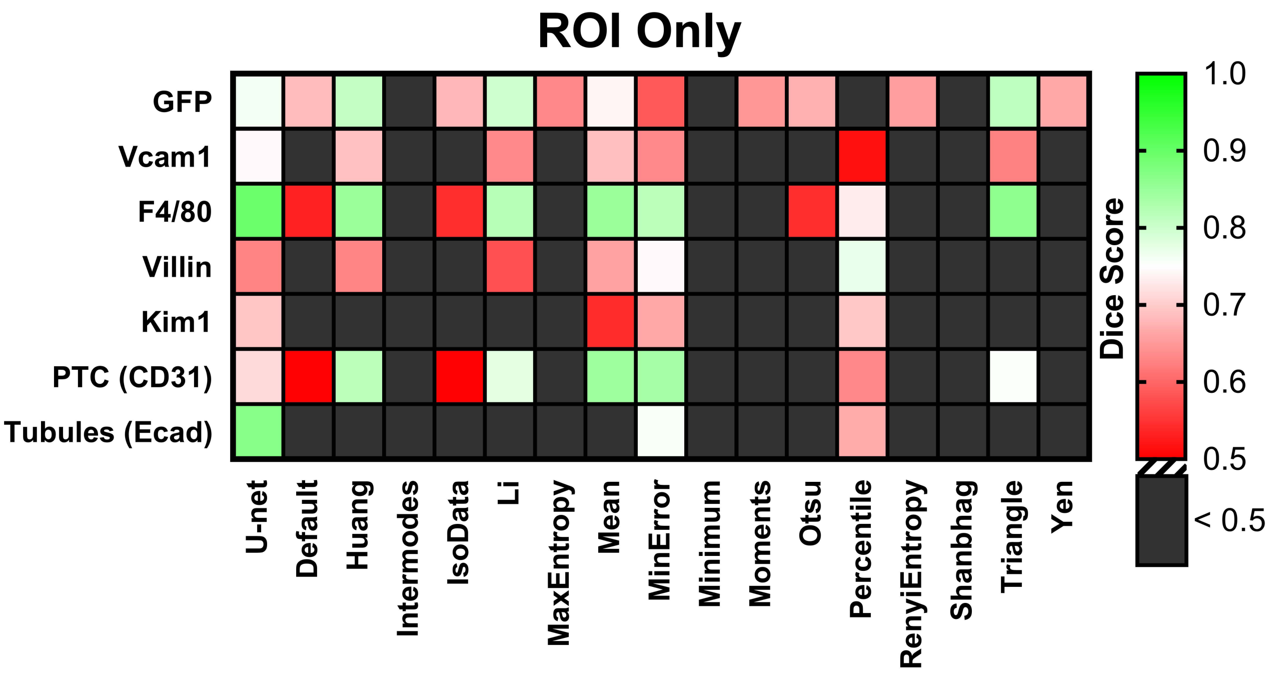

Supplementary Figure S4. Accuracy of ImageJ thresholding methods for membrane stains. The validation cohort of images were analyzed for each stain using all threshold methods available in ImageJ. Thresholding was either performed over the whole image and then extracted (top, Full Image) or thresholding was localized to each validation image separately (bottom, ROI Only). A heat map of the resulting Dice scores is shown. All Dice scores of less than 0.5 are shaded gray.

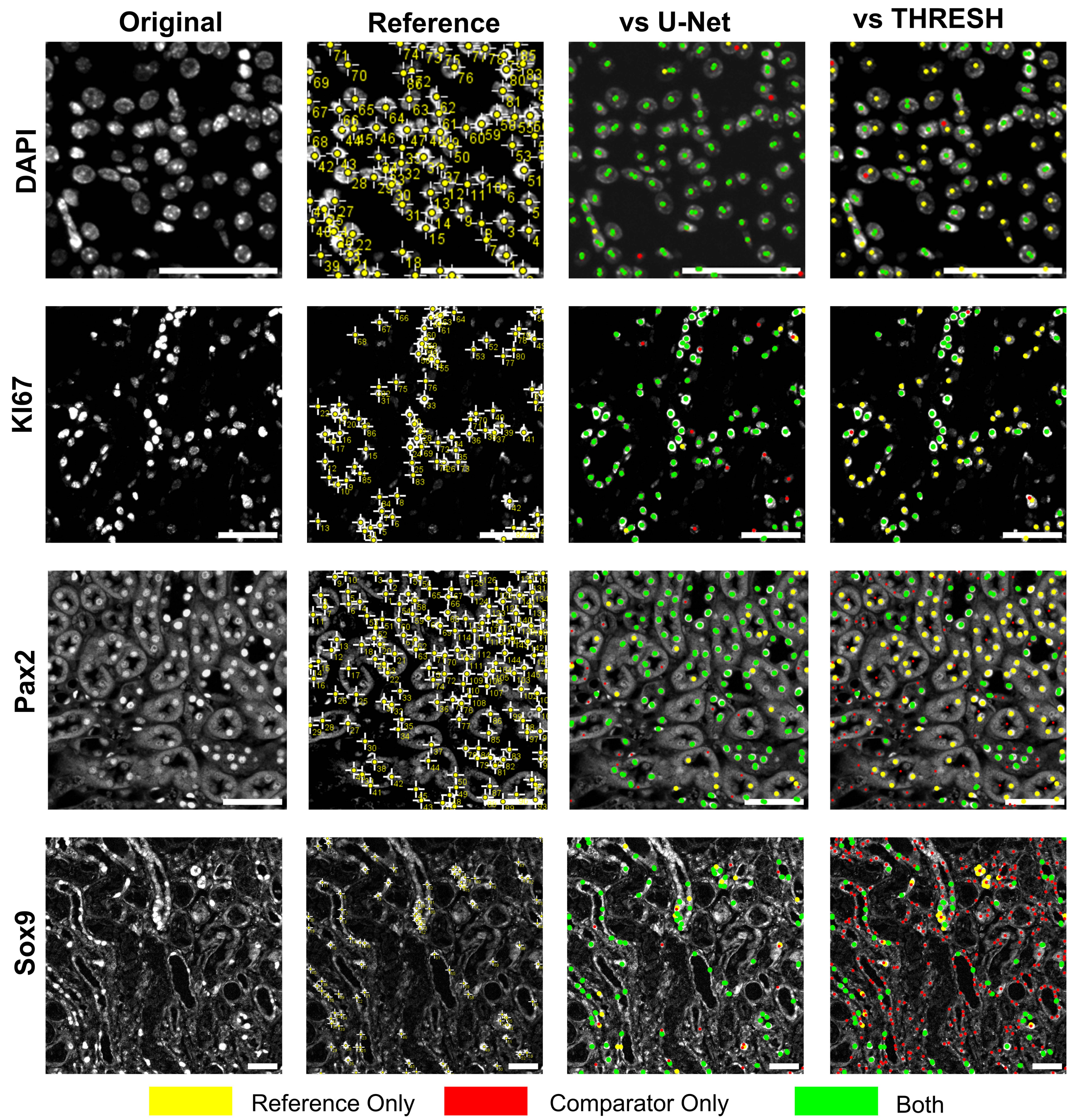

Supplementary Figure S5. Representative images of the nuclear stains showing manual annotations and comparisons between U-Net models and thresholding (THRESH). Nuclear coordinates were compared with the locations of nuclei marked by an expert reference annotator. Pairs of matching coordinates were determined (within 5 µm, only one match per annotation, marked by a pair of green dots linked by a green line). Scale bar = 50 µm.

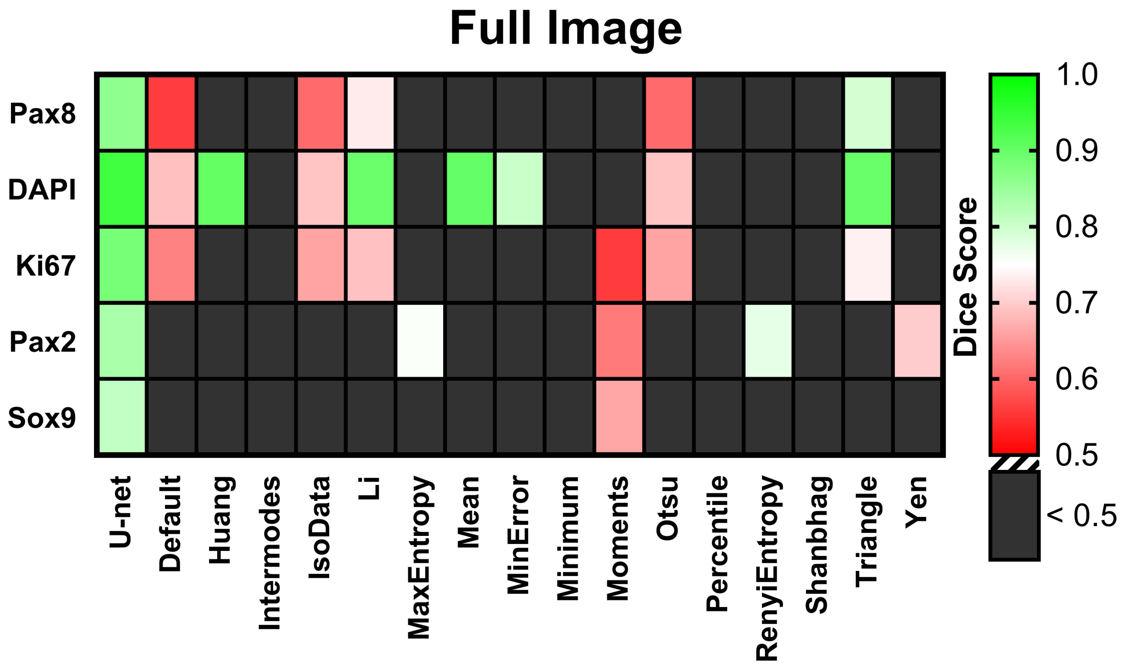

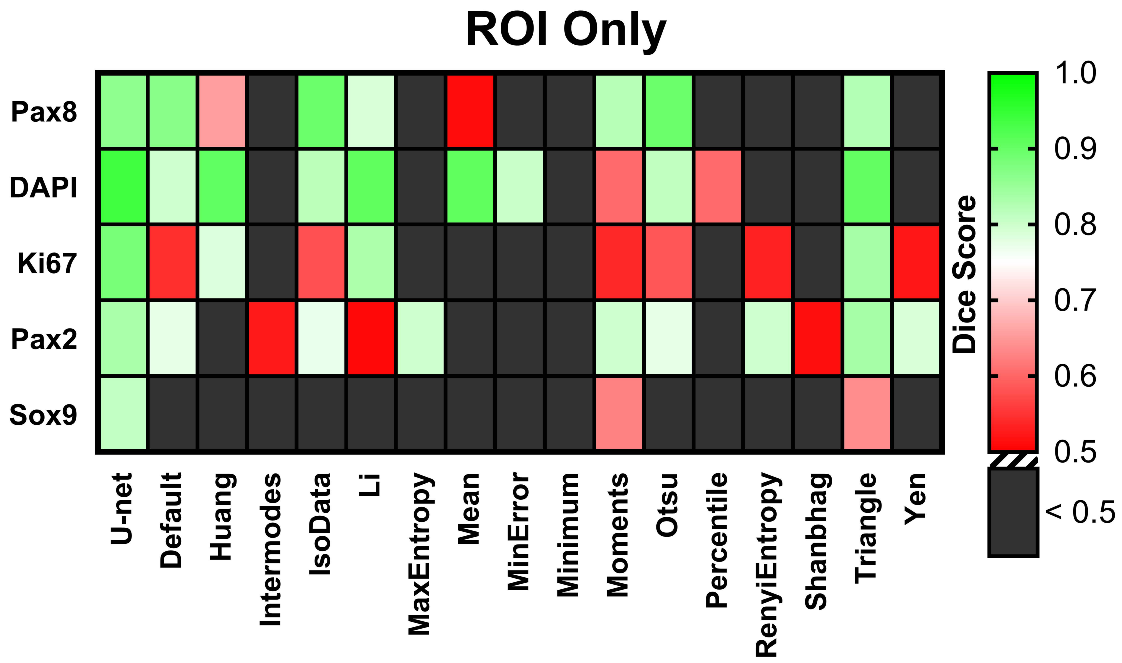

Supplementary Figure S6. Accuracy of ImageJ thresholding methods for nuclear stains. The validation cohort of images were analyzed for each stain using all threshold methods available in ImageJ, as in Supplementary Fig S2. Thresholding was either performed over the whole image and then extracted (top, Full Image) or thresholding was localized to each validation image separately (bottom, ROI Only). A heat map of the resulting Dice scores is shown. All Dice scores of less than 0.5 are shaded gray.

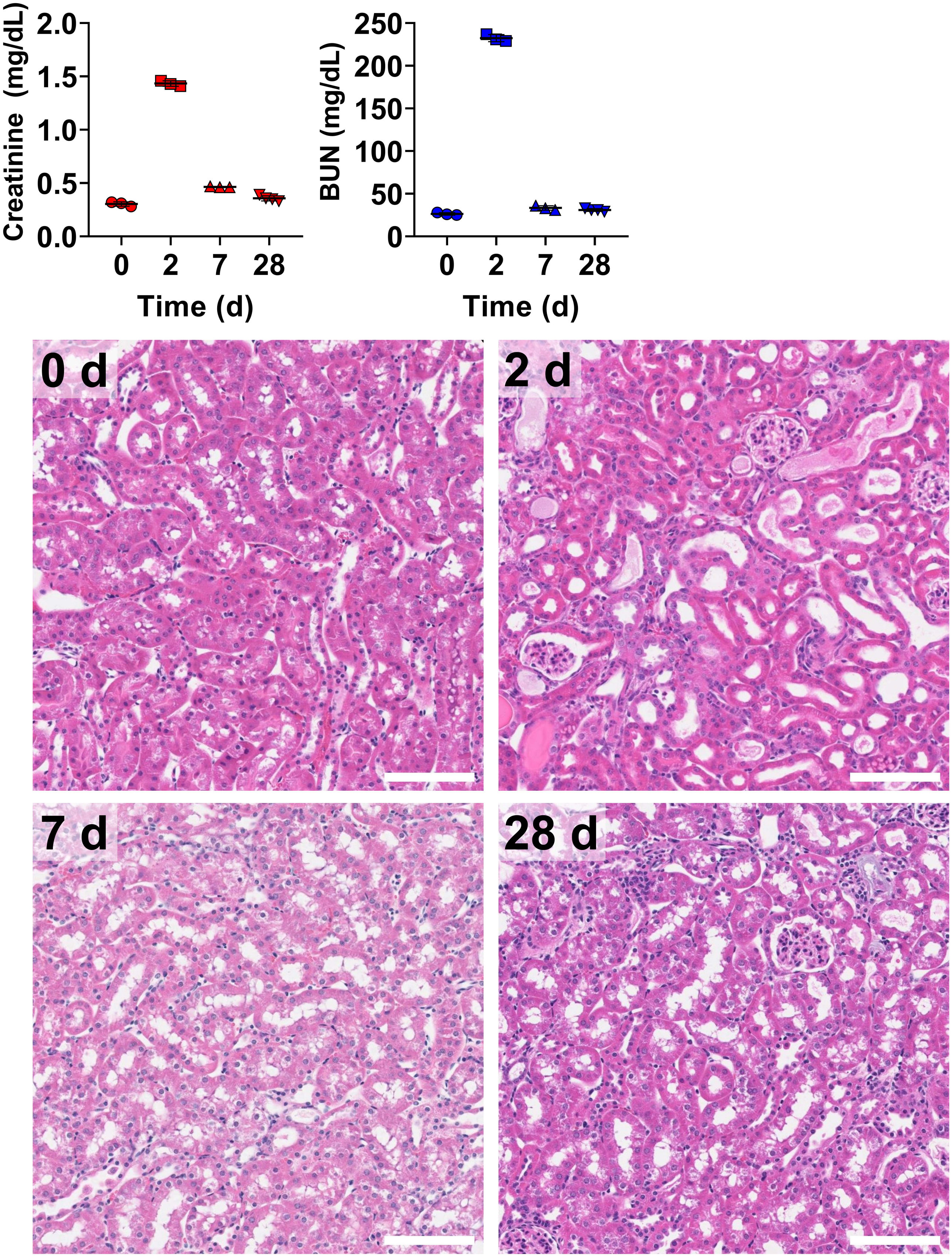

Supplementary Figure S7. Alternative assessments of injury in the folic acid nephrotoxicity model. Kidney function after folic acid nephrotoxicity over time as measured by serum creatinine, serum blood urea nitrogen (BUN) for animals analyzed in Fig. 4. Representative hematoxylin and eosin-stained sections from each time point are shown. Scale bar = 100 µm.

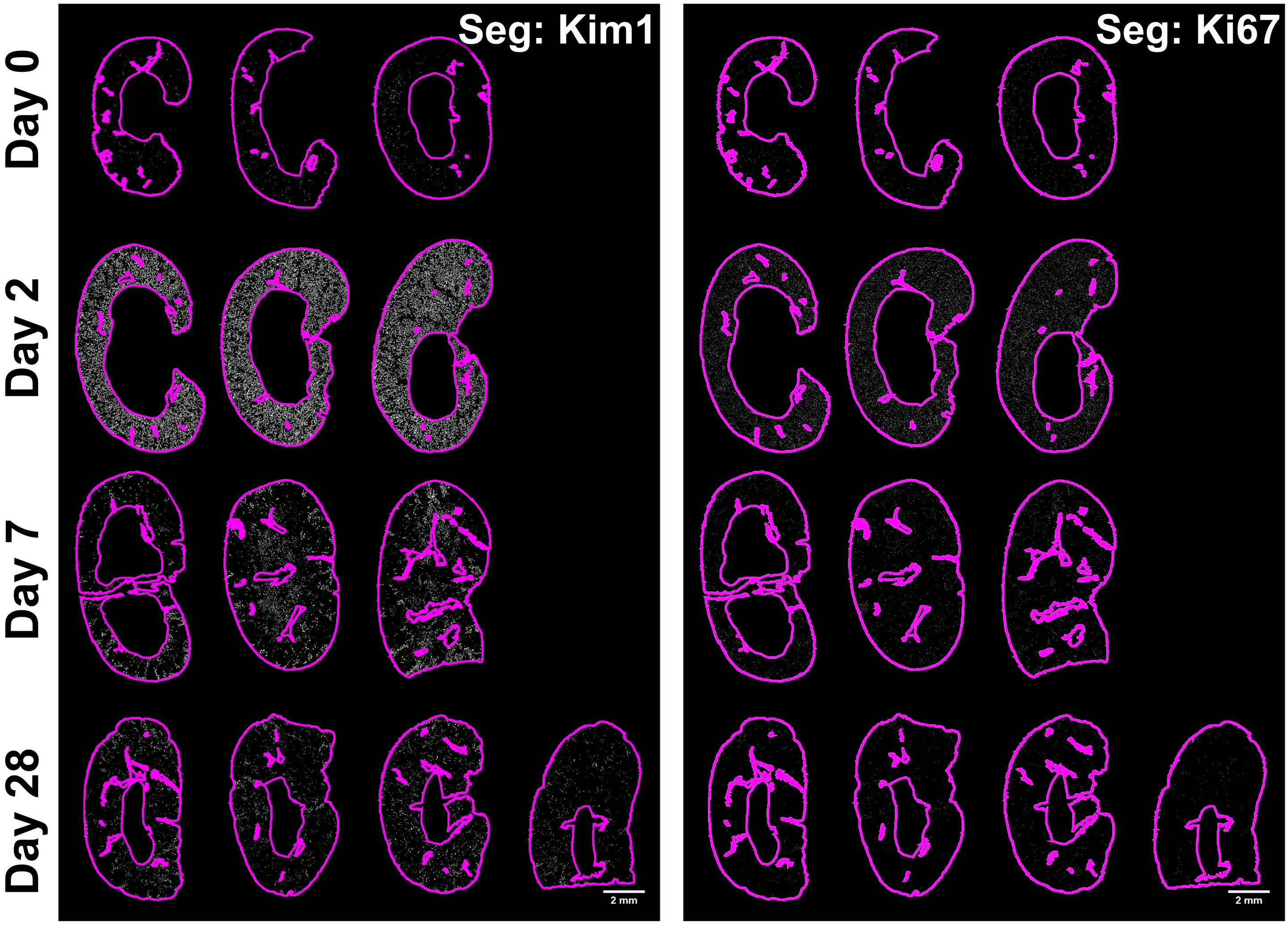

Supplementary Figure S8. Montages of segmentations for Kim1 (left) and Ki67 (right) for all animals/sections used for analysis. The cortex+OSOM is outlined in magenta. Scale bar = 2 mm.

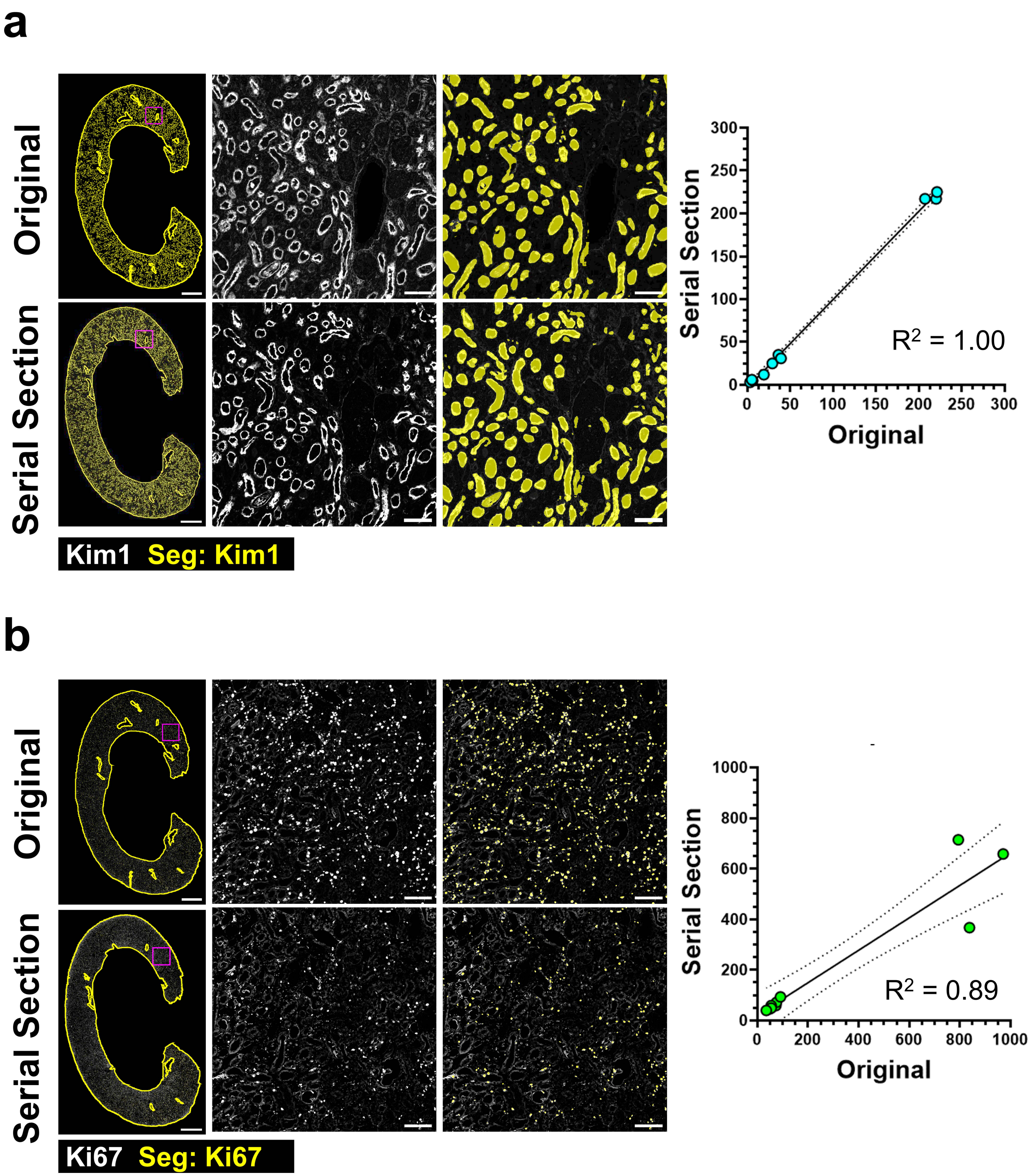

Supplementary Figure S9. Segmentation results are tightly correlated between serial sections from the same kidney. **a**) Two serial sections were stained for Kim1 and segmented using the corresponding U-Net models (shown as yellow overlay on the grayscale image). Results were analyzed for the cortex+OSOM of each section and compared (scatter plot, R^2 = 1.00). **b**) Density of Ki67 nuclei was also compared in a similar fashion (scatter plot, R^2 = 0.89). Note in the corresponding representative images (U-Net segmentation result shown as a yellow overlay) that differences were due to variation in the number of positive staining nuclei, not to segmentation accuracy. Some variation is expected for nuclear stains from section to section (~5 µm per slice), while larger structures will show high similarity. Dotted lines represent 95% confidence interval for linear regression. Scale bars: 1 mm (whole kidney), 100 µm (insets).

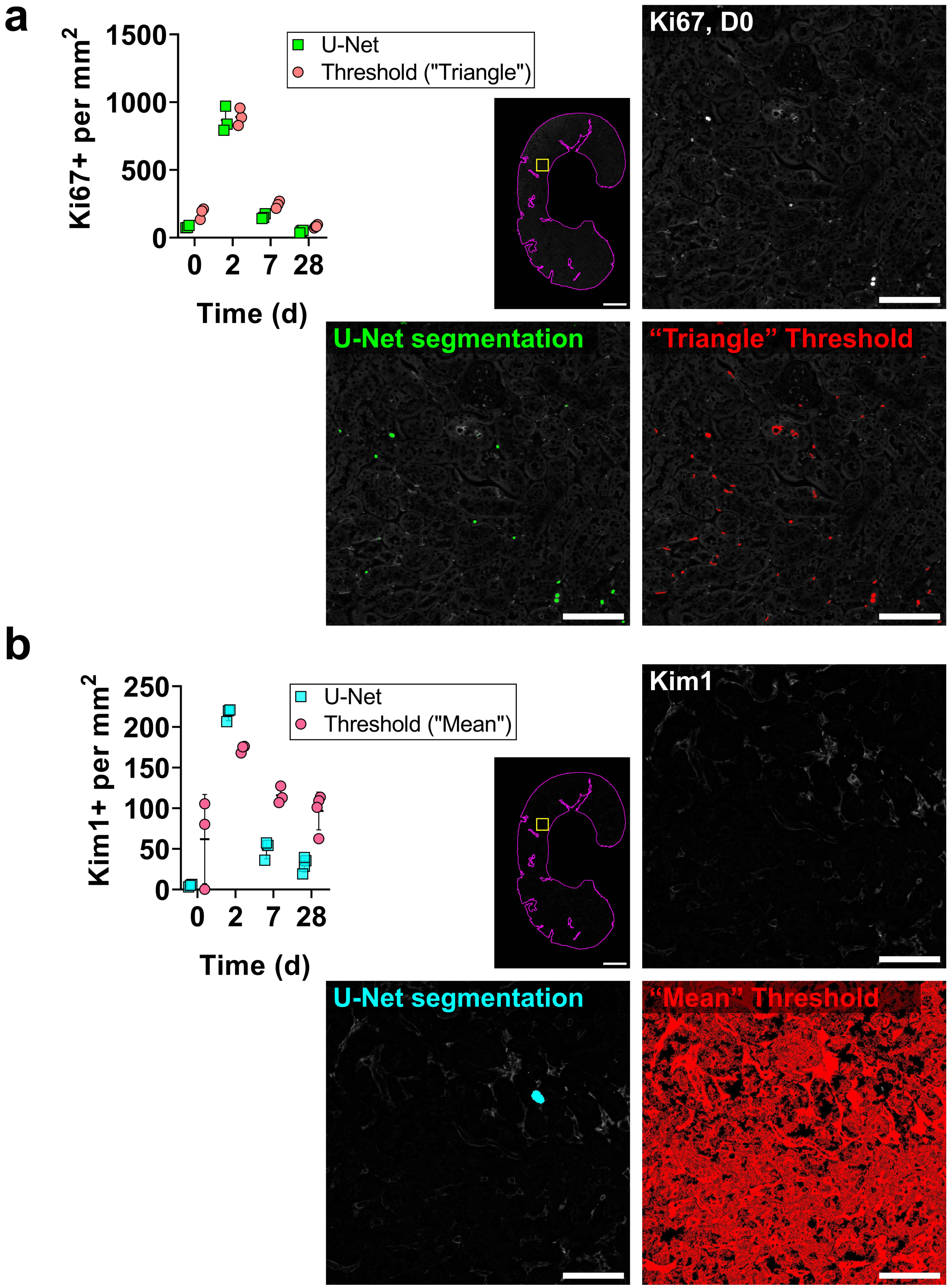

Supplementary Figure S10. Comparison of U-Net segmentations with optimized thresholding methods. **a**) Whole Ki67 images were analyzed using the "Triangle" thresholding method, which showed the highest Dice score in the validation cohort. The number of Ki67+ nuclei detected in the cortex + OSOM over time is shown as well as representative images comparing both methods at day 0. **b**) Whole Kim1 images were analyzed using the "Mean" thresholding method, which showed the highest Dice score in the validation cohort. The number of Kim1+ tubules detected in the cortex + OSOM over time is shown as well as representative images comparing both methods at day 0 in the same ROI as in (**a**). Note that Kim1 thresholding did not generalize from the validation cohort and grossly underperformed relative to U-Net. Scale bars: 1 mm (whole kidney), 100 μm (insets).

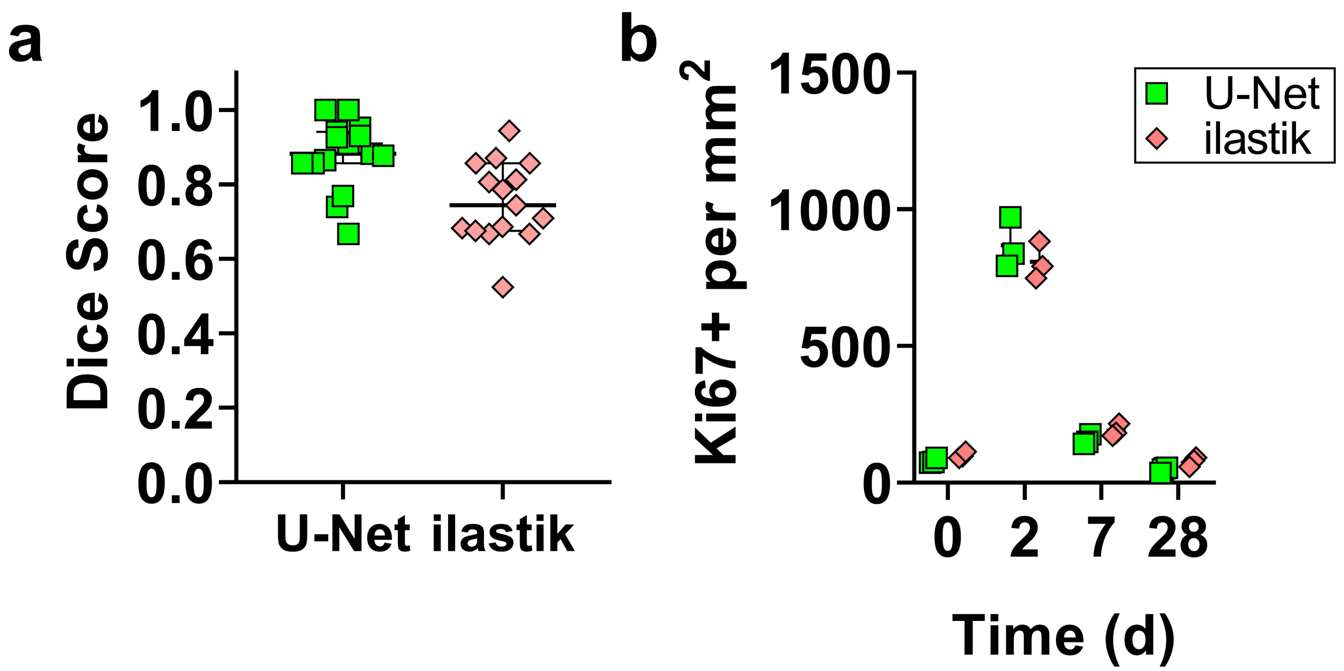

Supplementary Figure S11. Substitution of ilastik for U-Net. The tool ilastik was used to train a Random Forest machine learning pixel classifier to segment Ki67 positive nuclei. **a**) Segmentation performance was tested using the validation cohort of images used to generate Fig 3. **b**) The same whole kidney images used to generate Fig 4, were analyzed using ilastik, in place of U-Net, for segmentation and the resulting Ki67+ cells per area calculated and compared with those predicted using the U-Net model.

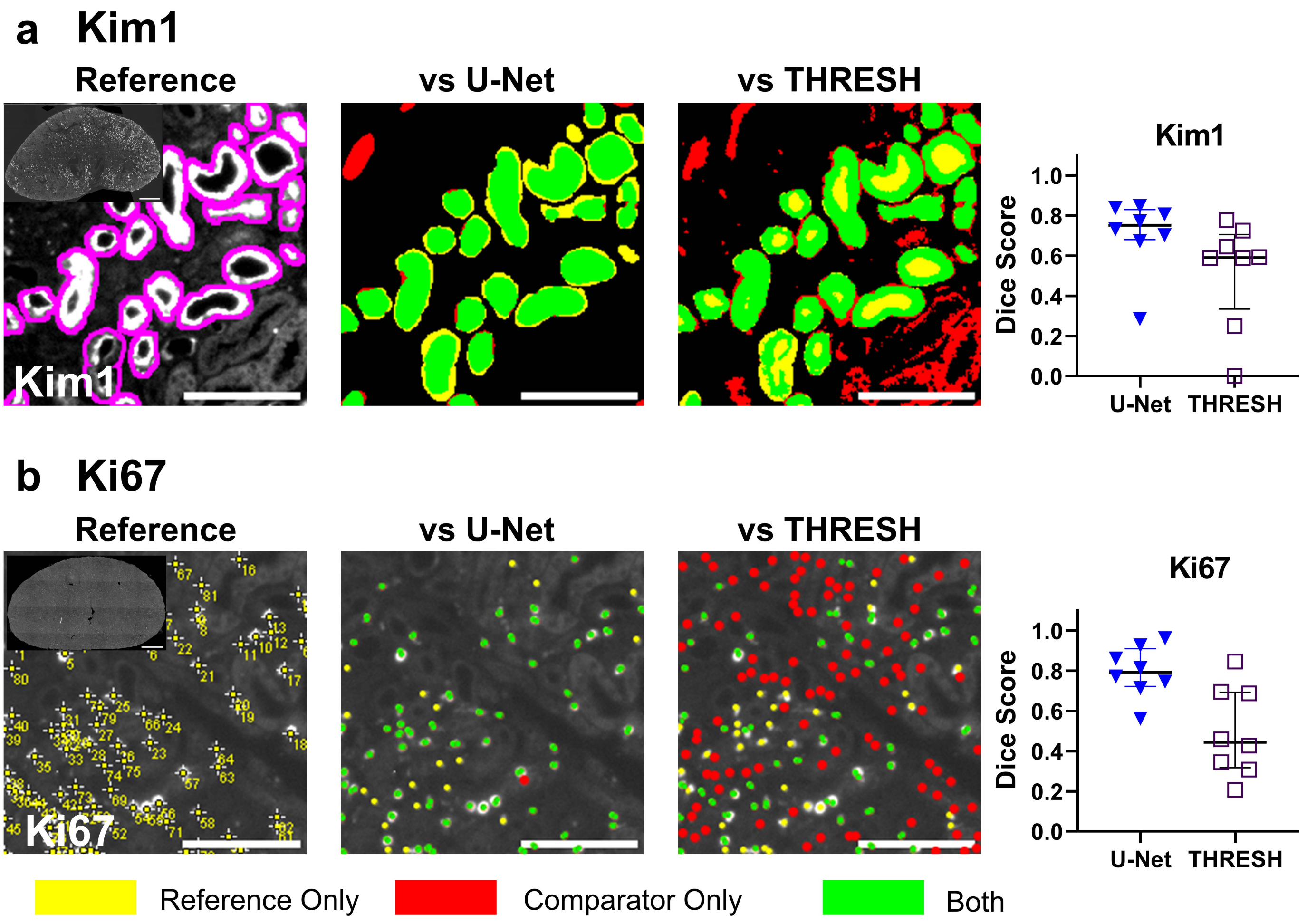

Supplementary Figure S12. Segmentation performance remains robust for images acquired with lower resolution. Images were acquired on a standard epifluorescence microscope with a motorized stage and stitched together with ImageJ. Whole sections were segmented using unadjusted U-Net models for Kim1 tubules and Ki67 or using thresholding (THRESH) of the background-subtracted image. A validation cohort of images was generated as in Figs 2 and 3 from injured kidney sections derived 14 d after ischemic injury (N = 8 animals). **a**) Representative images of Kim1 tubules showing reference annotations (magenta) and segmentation comparisons. Dice score for each image was calculated. Median + interquartile range are shown. **b**) Representative images of Ki67 staining with reference annotations and comparison to matched nuclei determined by U-Net segmentation or thresholding (THRESH). Matched points are shown as a pair of green dots linked by a green line. Dice score was calculated for each image. Median + interquartile range are shown. Scale bars: 1 mm (whole kidney), 50 μm (insets).

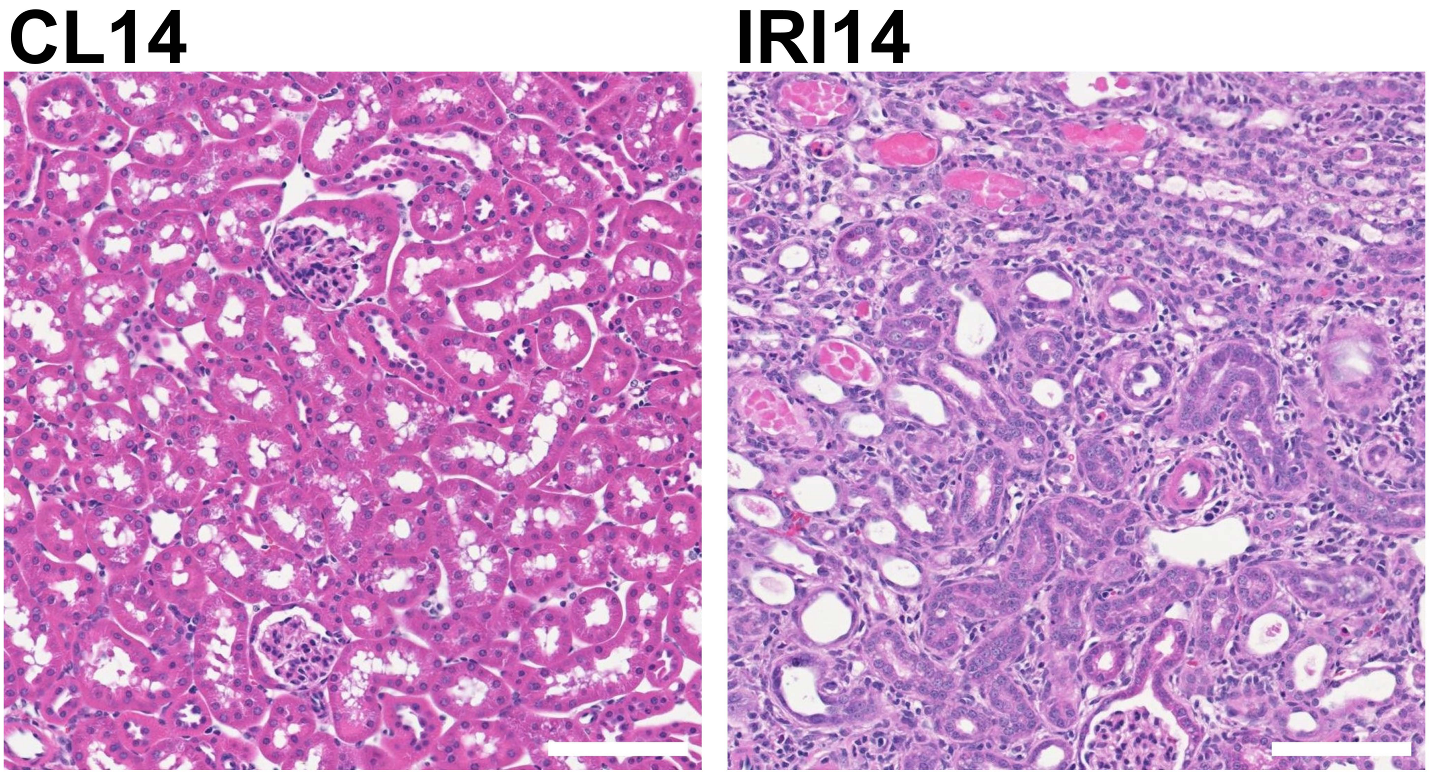

Supplementary Figure S13. Representative hematoxylin and eosin histology after severe unilateral ischemia reperfusion injury 14 d after injury in the contralateral uninjured kidney (CL14) and the ischemic kidney (IRI14). Scale bar = 100 μm.

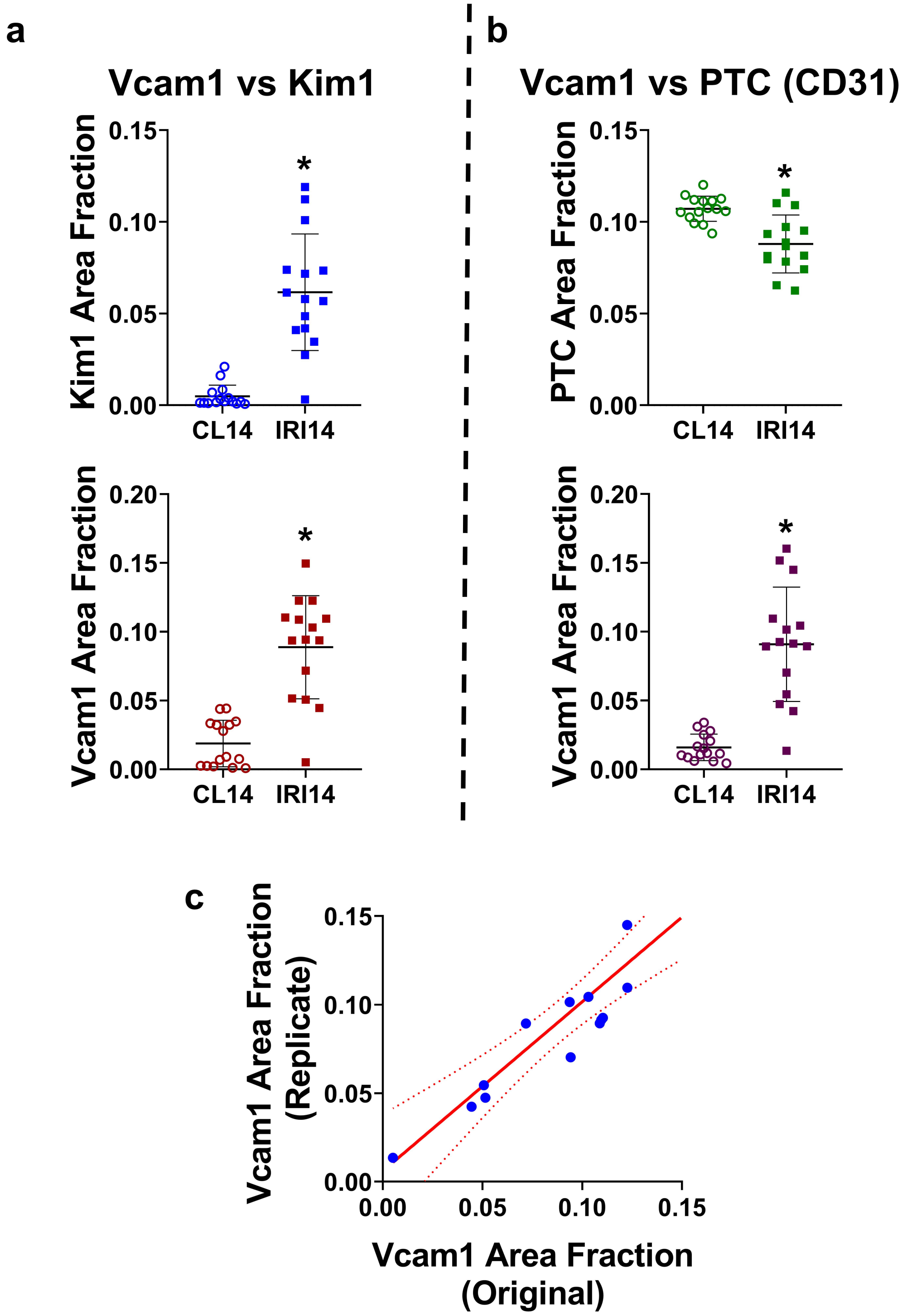

Supplementary Figure S14. Severe ischemic injury caused increased markers of failed repair and decreased peritubular capillary density 14 d after ischemic kidney injury. **a**) Whole sections of kidneys were segmented for Kim1+ tubules and Vcam1+ cells and area fraction of positive segmentation in the cortex + OSOM was calculated. The injured (IRI14) and contralateral uninjured (CL14) kidneys for each animal were compared. There were significantly more Kim1+ area ($p < 0.0001$, paired t-test) and Vcam1+ area ($p < 0.0001$, paired t-test). Note the heterogeneous response between animals (coefficient of variation 52% and 42% for Kim1 and Vcam1 respectively). **b**) Analysis of CD31 was performed on an independent set of non-sequential sections from the same animals as in panel (**a**). Conversely, there was a significant decrease in peritubular capillary density (PTC, marked by CD31) after injury ($p = 0.0003$, paired t-test). As with sections used in panel (**a**) there was also a significant increase in Vcam1 area after injury. Also note the heterogenous response (coefficient of variation 18% and 46% for PTC density and Vcam1 respectively). **c**) Vcam1 segmentation density was tightly correlated between animals on non-sequential sections (Pearson r = 0.86, $p < 0.0001$). Dotted lines represent 95% confidence interval for linear regression.

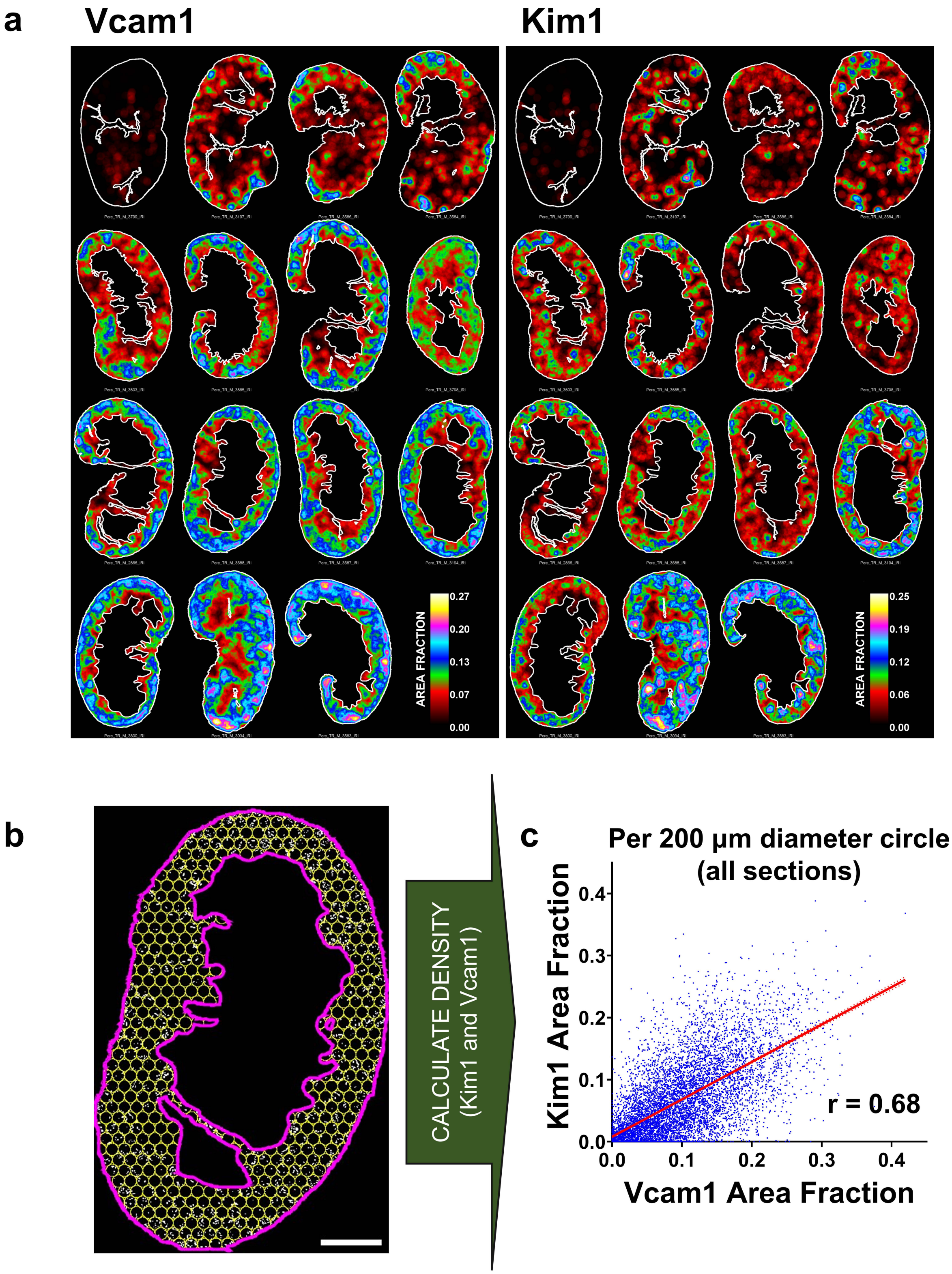

Supplementary Figure S15. Markers of failed repair after ischemic kidney injury are spatially correlated. Sections were stained for Vcam1 and Kim1, segmented using the corresponding U-Net models, and analyzed over the cortex + OSOM. **a**) Vcam1 and Kim1 segmentations were converted to a heatmap of area fraction (averaged over a 200 µm radius) and ordered by average area fraction per section for Vcam1. The analyzed cortex + OSOM is outlined in white. **b**) To assess local spatial correlation, the analyzed area of all sections was divided into close-packed non-overlapping circular ROIs. A representative section is shown with the analyzed area outlined in magenta and each ROI shown in yellow. **c**) The area fraction for Vcam1 and Kim1 for all ROIs across all samples (N = 8509) were compared and showed a positive correlation (Pearson r = 0.68, p < 0.0001). Scale bar = 1 mm.

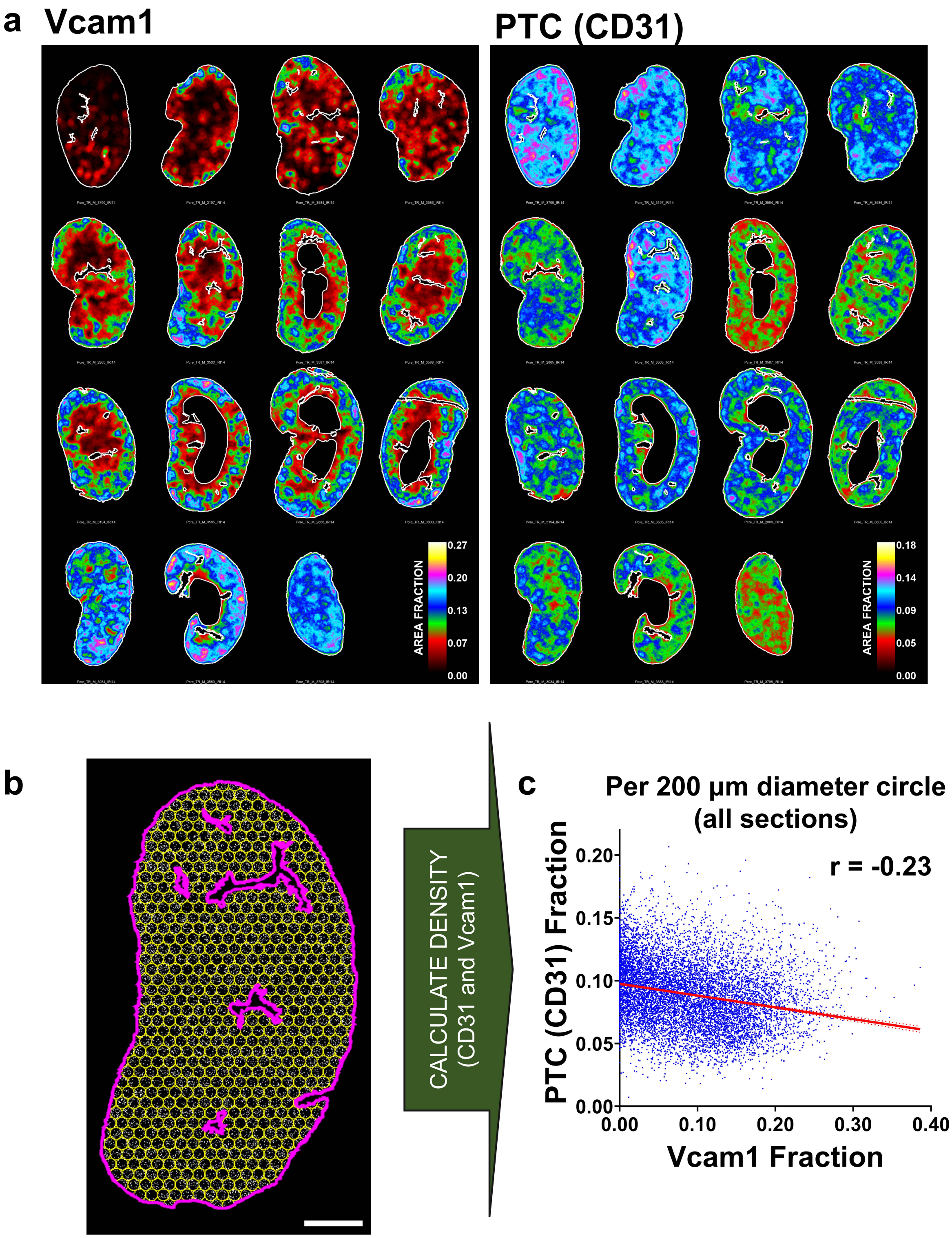

Supplementary Figure S16. Markers of failed repair after ischemic kidney injury are negatively spatially correlated with peritubular capillary density. Sections were stained for Vcam1 and CD31, segmented using the corresponding U-Net models for Vcam1 and peritubular capillaries (PTC) respectively, and analyzed over the cortex + OSOM. **a**) Vcam1 and PTC segmentations were converted to a heatmap of area fraction (averaged over a 200 µm radius) and ordered by average area fraction per section for Vcam1. The analyzed cortex + OSOM is outlined in white. **b**) To assess local spatial correlation, the analyzed area of all sections was divided into close-packed non-overlapping circular ROIs. A representative section is shown with the analyzed area outlined in magenta and each ROI shown in yellow. **c**) The area fraction for Vcam1 and PTCs for all ROIs across all samples (N =9877) were compared and showed a negative correlation (Pearson r = -0.23, $p <$ 0.0001). Scale bar = 1 mm.

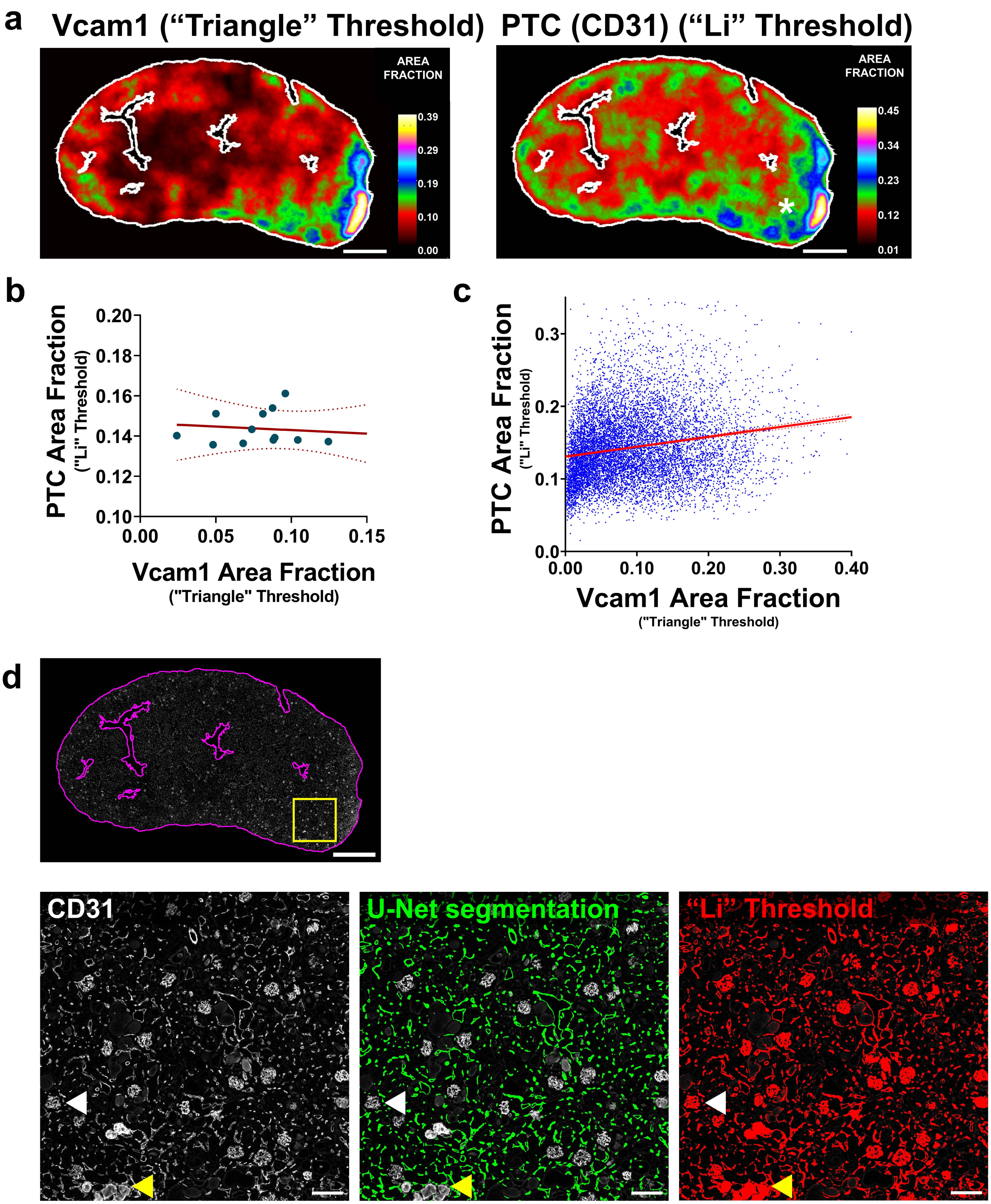

Supplementary Figure S17. Analysis of spatial correlation of Vcam1 and peritubular capillaries using thresholding. **a**) Whole kidney images used to generate Fig. 5c-d and Supplementary Fig. S16 were reanalyzed using the "Triangle" and "Li" thresholding methods for Vcam1 and CD31 stained peritubular capillaries, respectively. These methods showed the highest Dice score in the validation cohort for each stain (Supplementary Fig. S4). The results were used to produce heat maps of area fraction stained, as in Fig. 5 and Supplementary Fig. S16. Note that the area of reduced peritubular capillary density noted by the asterisk in Fig 5c is no longer apparent. **b**) Correlation, by animal, observed with U-Net analysis was not observed using thresholding. **c**) Spatial correlation, which was modestly negative with U-Net, was positive using thresholding. **d**) Inspection of images revealed numerous false positive detections of glomerular capillaries (white arrowhead) and debris (yellow arrowhead). ROI shown corresponds to region in asterisk in Fig 5c. Scale bars: 1 mm (whole kidney), 100 μm (insets).

Supplementary Table S1: Antibodies

Target	Supplier	Product Number	Dilution
CD31	Biotechne	AF3628	1/200
E-Cadherin	BD - Biosciences	610182	1/800
F4/80	IONPATH	D259R	1/400
GFP	Invitrogen	A10262	1/400
Ki67	Biolegend	652402	1/200
Kim1	Biotechne	AF1817	1/200
Pax2	in house[48]		1/3200
Pax8	Proteintech	10336-1-AP	1/400
Sox9	Biotechne	AF3075	1/400
Vcam1	Abcam	ab134047	1/400
Villin	Santa Cruz	SC-7672	1/200

[48] Dressler, G. R. & Douglass, E. C. Pax-2 is a DNA-binding protein expressed in embryonic kidney and Wilms tumor. Proc Natl Acad Sci U S A 89, 1179-1183 (1992). https://doi.org:10.1073/pnas.89.4.1179
